# The economic evaluation of human papillomavirus vaccination strategies against cervical cancer in women in Lao PDR: a mathematical modelling approach

**DOI:** 10.1186/s12913-016-1662-5

**Published:** 2016-08-22

**Authors:** Phetsavanh Chanthavilay, Daniel Reinharz, Mayfong Mayxay, Keokedthong Phongsavan, Donald E. Marsden, Lynne Moore, Lisa J. White

**Affiliations:** 1 Faculty of Postgraduate Studies, University of Health Sciences, Vientiane, Lao PDR; 2 Mahidol-Oxford Tropical Medicine Research Unit, Faculty of Tropical Medicine, Mahidol University, 420/6 Rajvithi Road, Bangkok, 10400 Thailand; 3 Department of Social and Preventive Medicine, Faculty of Medicine, Laval University, 1050, avenue de la Médecine, Quebec, QC G1V 0A6 Quebec Canada; 4 Institut de la Francophonie pour la Médecine tropicale, Vientiane, Lao PDR; 5 Lao-Oxford-Mahosot Hospital-Wellcome Trust Research Unit (LOMWRU), Microbiology Laboratory, Mahosot Hospital, Samsenthai Road, P.O. Box 744, Vientiane, Lao PDR; 6 Centre for Tropical Medicine and Global Health, Churchill Hospital, University of Oxford, Oxford, UK; 7 Gynecologic Oncology Unit, Setthathirath Hospital, Ban Donekoi, Sisatthanak Districk, P.O.Box: 6088, Vientiane, Lao PDR; 8 Nuffield Department of Medicine, University of Oxford, Oxford, UK

**Keywords:** Economic evaluation, HPV vaccination, Cervical cancer, Lao PDR

## Abstract

**Background:**

Cervical cancer, a preventable disease, is the third leading cause of cancer morbidity and mortality in the Lao People’s Democratic Republic (Lao PDR). Since many cervical cancers are linked to human papilloma virus (HPV) infection, vaccination against this virus may lead to a reduction in these types of cancer. The study described here is the first to compare the cost-effectiveness of different HPV vaccination options in Lao PDR.

**Methods:**

A dynamic compartment model was created. The model included routine screening activities already in place, as well as theoretical interventions that included a 10-year old girl-only vaccination programme combined with/without a 10-year old boy vaccination programme and/or a catch-up component. The simulation was run over 100 years. In base case analyses, we assumed 70 % vaccination coverage with lifelong protection and 100 % efficacy against HPV types 16/18. The outcomes of interest were the incremental cost per Disability-Adjusted Life Year (DALY) averted.

**Results:**

In base case analyses, according to the WHO definition of cost-effectiveness thresholds, vaccinating 10-year-old girls was very cost-effective. Adding a catch-up vaccination element for females aged 11–25 years was also very cost-effective, costing 1559 international dollars (I$) per DALY averted. Increasing the age limit of the catch-up vaccination component to 75 years old showed that this remained a cost-effective option (I$ 5840 per DALY averted). Adding a vaccination programme for 10-year-old boys was not found to be cost-effective unless a short time simulation (30 years or less) was considered, along with a catch-up vaccination component for both males and females.

**Conclusions:**

Adding a catch-up female vaccination component is more attractive than adding a 10-year-old boy vaccination component.

**Electronic supplementary material:**

The online version of this article (doi:10.1186/s12913-016-1662-5) contains supplementary material, which is available to authorized users.

## Background

Cervical cancer is the third leading cause of cancer deaths among women in Lao People’s Democratic Republic (Lao PDR), with an estimated crude incidence and mortality rates of 9.8 and 5.3 per 100,000 women annually [1]. The vast majority of cervical cancers are caused by infection with human papillomaviruses (HPV), particularly HPV types 16 and 18. The high fatality rate associated with cervical cancer in Lao PDR is probably due to various factors. This includes the lack of a national HPV vaccination programme, lack of effective chemo/radiotherapy in the country, and delays in diagnosis [1]. The delay in diagnosis is in great part due to the fact that there is no national cervical cancer-screening programme in Lao PDR, where it has been estimated that only 5 % of females aged 18–69 in urban areas and 1 % in rural areas are screened every 3 years [1].

A systematic screening programme might reduce the disease burden, but may not be possible in Lao PDR due to various reasons including financial and sociocultural barriers, poor healthcare infrastructure, and poor performance of laboratory tests [2]. Given these problems, HPV vaccination might be a more suitable approach for the country. It has been shown to be efficacious, with bivalent and quadrivalent vaccines providing extremely high rates of protection against high grade cervical intraepithelial neoplasia (CIN 2/3) related to HPV types 16 and 18 [3]. Moreover, Goldie and colleagues [4] showed that HPV vaccination in preadolescent females is very cost-effective in 72 Global Alliance for Vaccines and Immunization (GAVI)-eligible countries, including Lao PDR, and Jit and colleagues [5] found similar outcomes on a global scale. However, no nationwide vaccination strategy has so far been implemented in Lao PDR.

A HPV vaccination pilot project, which consists of vaccinating 5^th^ grade schoolgirls (10–13 years old) in the Lao capital Vientiane, and in neighbouring Vientiane Province, is currently taking place. It is likely that such a HPV vaccination programme will become routine practice in the future. However, vaccination coverage might be low. Considering this eventuality, the authors thought it is important to evaluate the benefit of complementing such a vaccination programme with additional interventions, such as adding a catch up vaccination campaign and/or a 10-year-old boy vaccination element. In order to examine these questions, we used a mathematical modelling approach to estimate the cost-effectiveness of various HPV vaccination strategies in the Lao context.

## Methods

### Model structure

Inspired by previous economic models of HPV vaccination [6], a compartmental dynamic population-based model was created to reflect the expected effect of HPV vaccination programmes, both in females and males. The model considered whether the HPV genotypes were 16, 18 or other high-risk type, or a low-risk type. (Full methodological details are provided in the Additional file 1).

**Fig. 1:**
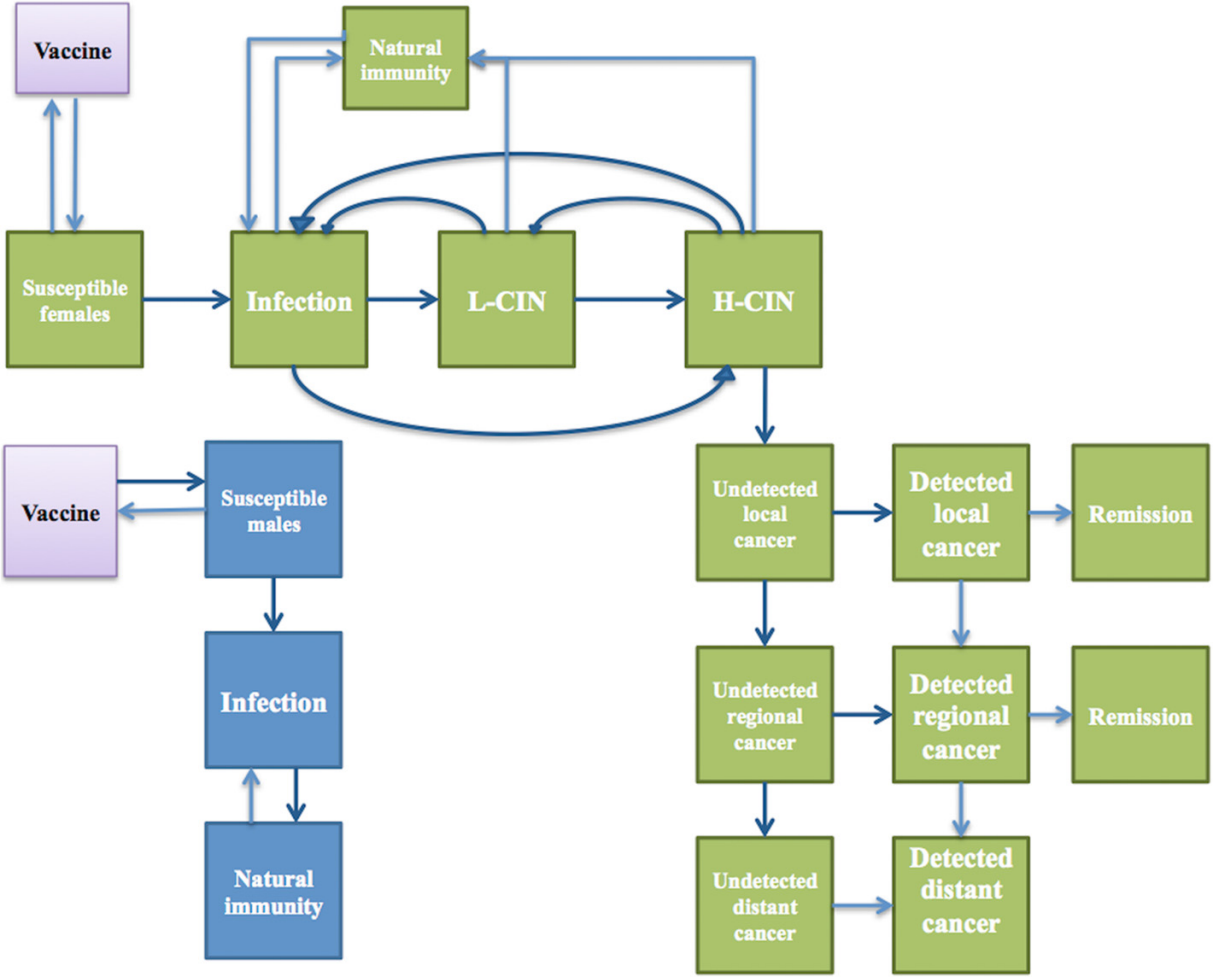
Model structure for the natural history of human papillomavirus infection and cervical cancer. The model structure reflects the natural history of HPV infection towards cervical cancer. Women can be infected by HPV and progress to low-grade CIN or high-grade CIN, or regress with natural immunity. Low-grade CIN progress to high-grade CIN, or regress thanks to the natural immunity. High-grade CIN progress to invasive cervical cancer (local, regional and distant cancer), or regress thanks to the natural immunity. In the male model, there are three compartments considered: susceptibility to infection, infection and recovery with natural immunity. Female can be protected by HPV vaccine

For females, the model considered that an infection with HPV regresses due to natural immunity, while remaining susceptible to infection with other HPV types. HPV infection may persist or progress into a cervical intraepithelial neoplasia (low-grade CIN or high-grade CIN). A low-grade CIN might regress to an immunity state or an infection state, or progress into a high-grade CIN. A high-grade CIN might regress to an immunity state or an infection state or low-grade CIN, or might progress into an invasive cervical cancer (local, regional or distant, respectively). Women diagnosed with a high-grade CIN are treated. Women with invasive cervical cancer might be symptomatically detected. Diagnosed cancer cases are treated, with a probability of recovery, treatment failure or death (Fig. 1).

For males, only the susceptibility, infection and recovery states were considered. Vaccinated people remained susceptible for non-vaccine HPV types.

### Parameters

Monthly age-specific transition probabilities from one lesion state to another and regression rates presented in Table 1 were taken from Kim and colleagues [7], while infection rates were calculated by multiplying the sexual relationship matrix by HPV genotype-specific transmissibility and age-specific HPV prevalence in the opposite sex. To simplify the model, we considered all members of the population to be heterosexual. The sexual relationship matrix consisted of the monthly age-specific probability of having a new sexual partner, in which each age group has the probability of having sexual intercourse with someone of the same and a different age group of 0.6 and 0.4 respectively [8] (See Additional file 1). The initial age of sexual intercourse was taken to be 15 years old or more in both females and males [9]. The screening and treatment parameters are also described in the Additional file 1.

**Table 1:** Summary of input parameters for the model

Parameters		Baseline values^a^	Range^b^	Source
Progression				
Healthy to infection^c^	HPV-16	0.000175–0.003148	0.0001426–0.00761	Calibrated
	HPV-18	0.0004–0.000789	0.000102–0.00168	
	Other HR HPV	0.000206–0.004038	0.0001703–0.00911	
	LR HPV	0.000958–0.018412	0.00069–0.0537	
HPV DNA to CIN1^d^	HR-16 HPV	0.005194–0.00901		[7]
	HR-18 HPV	0.002793–0.004845		
	HR-other HPV	0.007693–0.013345		
	LR-HPV	0.002397–0.001222		
Proportion (%) of women who transition directly from HPV DNA to CIN2,3	HR-16 HPV	0.64		[7]
	HR-18 HPV	0.975		
	HR-other HPV	0.966		
	LR-HPV	0.98		
CIN 1 to CIN 2,3^d^	HR-16 HPV	0.00951–0.012363		[7]
	HR-18 HPV	0.0051–0.00663		
	HR-other HPV	0.00747–0.009711		
	LR-HPV	0.000149–0.000222		
CIN 2,3 to local cancer	HR-16 HPV	0.000151–0.00906		[7]
	HR-18 HPV	0.000264–0.01584		
	HR-other HPV	0.000199–0.01194		
Local to regional invasive cancer		0.0200		[7]
Regional to distant invasive cancer		0.0250		
Regression				
HPV DNA to normal	HR-16 HPV	0.09089		[7]
	HR-18 HPV	0.09089		
	HR-other HPV	0.09272		
	LR-HPV	0.09699		
CIN 1 to normal^e^	HR-16 HPV	0.03782		[7]
	HR-18 HPV	0.03782		
	HR-other HPV	0.04575		
	LR-HPV	0.01708		
CIN 2,3 to normal^f^	HR-16 HPV	0.000798–0.000455		[7]
	HR-18 HPV	0.003556–0.011938		
	HR-other HPV	0.002926–0.009823		
	LR-HPV	0.001904–0.006392		
Other				
Immunity (%) (HR-HPV types only)^g^	HR-16 HPV	0.66		[7]
	HR-18 HPV	0.86		
	HR-other HPV	0.59		
Annual probability of symptom detection^h^	Local invasive cancer	0.33		[7]
	Regional invasive cancer	0.60		
	Distant cancer	0.9		
Proportion of cancer patient receiving the treatment	Local cancer	100 %		Calibrated
	Regional cancer	80 %		
	Distant cancer	70 %		
Age-specific 5-year survival proportion after diagnosis and treatment (%)^i^	Local cancer	0.29–71 %		Calibrated
	Regional cancer	0.24–78 %		
Age-specific monthly probability of death	Complication of local cancer treatment	0.012–0.037		Calibrated
	Complication of regional cancer treatment	0.0098–0.028		
	Distant cancer	0.28–0.83		

Note:

^a^Baseline values are monthly age-specific rates, unless otherwise noted

^b^Range is age-specific rate calibrated with the assumption of unchanged natural progression and regression of HPV infection and cervical cancer

^c^The transition from healthy state to infection is a force of infection derived from the number of sexual partner change, HPV type-specific transmissibility (range: 0.353–0.41)

^d^ *HPV* human papillomavirus, *CIN* cervical intraepithelial neoplasia, *HR* high risk, *LR* low risk

^e^70 % of women with CIN 1 regress to normal, 30 % to HPV

^f^70 % of women with CIN2,3 regress to normal, 15 % to HPV, 15 % to CIN 1

^g^Immunity represents the degree to protection each woman faces against future type-specific infection after infection after first infection and clearance. The immunity was assumed to be lifelong protection

^h^The annual probability of symptom detection corresponds to 15 % for local cancer and 85 % for advanced cancer

^i^Age-specific survival proportion was calibrate, based on a mortality rate by Globocan [1]

### Model calibration

The population was stratified by gender and age. The model is in the form of a realistic age structured (RAS) model. The equations were numerically solved in Berkeley Madonna version 8.3.18 [10]. The model was calibrated using maximum likelihood. The details are described in the Additional file 1. Briefly, the model was first calibrated in order to produce a demographic structure similar to the 2014 distribution of the Vientiane Capital province population (in one-year intervals) [11]. Thereafter, the model was calibrated for the age-specific incidences and mortalities of cervical cancer, according to the Globocan estimates [1].

### Scenarios

The baseline model considered that in Lao PDR there is no vaccination program, and that the coverage of routine cytology screening is 5.2 % among females aged 18–68 [1]. We assumed that screening coverage would remain the same over time. The HPV vaccination programmes investigated consisted of a 10-year-old girl vaccination programme alone, or combined with a catch-up component and/or a 10-year-old boy vaccination element. The details of the strategies are summarised in Table 2. The population of 10-year-old girls was chosen because the current HPV vaccination pilot project targets 5^th^ grade female students who are mostly 10 years old. The first selected age group for a catch-up vaccination in females was 11–25 year-olds, as this age group represents female undergraduate students who are reachable through school and university-based interventions. The 11–75 year-old age group represents the population at risk of HPV infection in our model.

**Table 2:** Summary of the vaccination strategies evaluated

	Female			Male		
	Routine vaccination	Catch up campaign		Routine vaccination	Catch up campaign	
	10 years	11–25 years	11–75 years	10 years	11–25 years	11–75 years
1	X					
2	X			X		
3	X	X				
4	X	X		X		
5	X	X		X	X	
6	X		X			
7	X		X	X		
8	X		X	X		X

Note: Routine vaccination is to give HPV vaccine to a 10-year-old girl and/or boy every year

Catch-up campaign is one-year catch-up HPV vaccination for females and/or male aged 11-25 years or 11-75 years

The coverage of HPV vaccination was assumed to be about 70 % (30–80 %), with 100 % (30–100 %) effectiveness against HPV type 16 and 18 and a lifelong protection (10 years to lifelong).

### Costing

The perspective considered was essentially that of the public healthcare system. Only direct medical costs and the programmatic cost of the vaccination programme itself were considered. The costing methodology is detailed in the Additional file 1. Briefly, the cost of delivering HPV vaccines consisted of the price of the vaccine and the programmatic cost of vaccination delivery. The programmatic cost of a three-dose HPV vaccine per individual was retrieved from an evaluation on HPV vaccination performed in Vientiane by the WHO (Phanmanysone Philakong, personal communication). The Global Alliance for Vaccines and Immunization (GAVI) vaccine cost per dose was used [12]. Medical costs were estimated based on data from a cost study done by the Lao PDR Ministry of Health (Maytry Senchanthixay, personal communication). This included cytology screening, visits & examinations, laboratory tests, and treatments for precancerous lesions and cervical cancer. The cost of treatment for stage-specific invasive cervical cancer was obtained from Goldie and colleague [4]. Unit prices are reported in the value of 2013 international dollars (I$), using purchasing power parity (PPP).

### Analyses

The simulation process was run deterministically over a 100-year span to capture the long-term benefits of vaccination. For each option, the output consisted of the cumulative number of cervical cancers per 1000 women, the Disability-adjusted life years (DALYs) per 1000 women, and the cost of screening and treatment per 1000 women. The strategies were ranked based on their cost, from lowest to highest. In the case of a non-dominant situation, strong, or extended dominance, the incremental cost-effectiveness ratio (ICER) was calculated using the reduction of HPV type 16/18-related cervical cancer cases and DALYs averted as denominators. DALYs were calculated based on the Global Burden of Disease, using standard life expectancy, without age weighting [13]. The disability weighting for cancer treatment was retrieved from current literature [14]. All costs and DALYs were discounted at a rate of 3 % in the base case simulations [15]. The cost-effectiveness results were organised into three categories: 1) very cost-effective (ICER < 1 Lao gross domestic product (GDP) per capita; 2) cost-effective (ICER between 1 and 3 times the GDP per capita); and 3) not cost-effective (ICER > 3 times the GDP per capita) [16]. One-way sensitivity analyses were conducted to identify the parameters, according to the literature and assumption, that might influence the incremental cost-effectiveness ratio per DALY averted.

## Results

### Model calibration

**Fig. 2:**
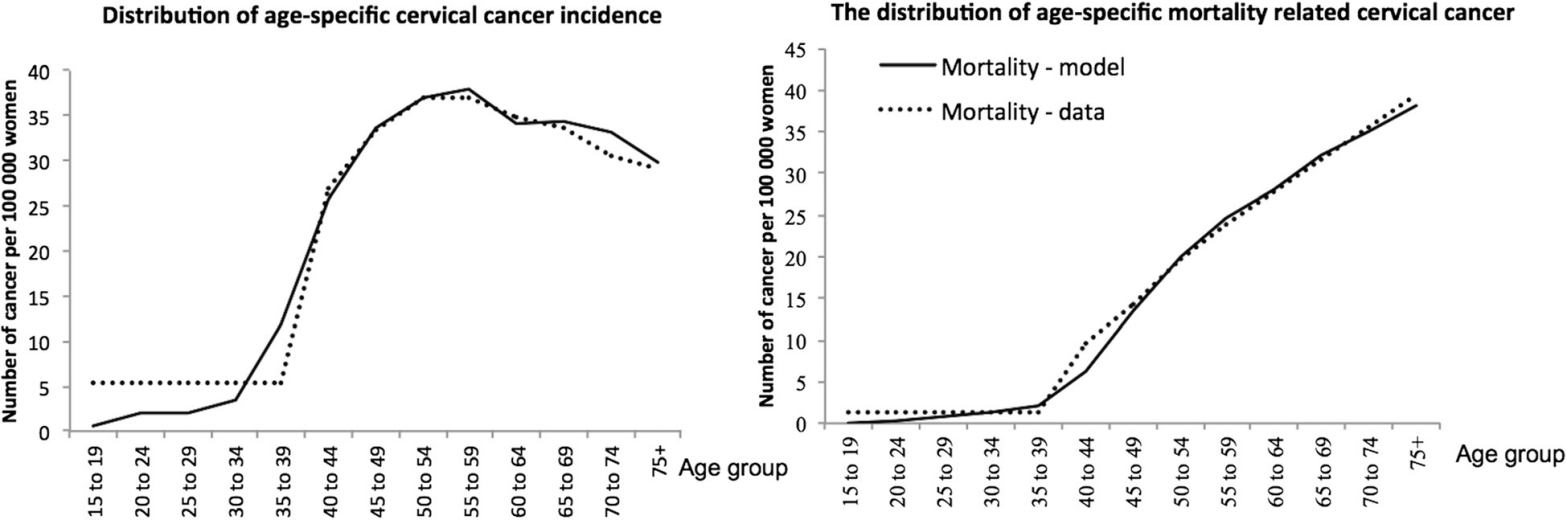
Model calibration to age-specific incidence and mortality of cervical cancer. Predicted incidence of cervical cancer and predicted mortality related to cervical cancer follow the age specific-distribution of observed data in Lao PDR, as estimated by Glocoban

The model was able to reproduce the expected values regarding demographic data in 2014 for Vientiane Capital, for both female and male populations (Additional file 1). However, the number of individuals was high for those aged 10–25 years compared with expected values, while it was low for those aged 25–35 years old. The model reproduced results for the estimated incidence of cervical cancer and its mortality due to any high-risk HPV type that were consistent with the estimates of Globocan, 2012 (Fig. 2). The proportion of cervical cancers related to HPV types 16 and 18 was about 75 %.

### Clinical impact and cost-effectiveness

**Fig. 3:**
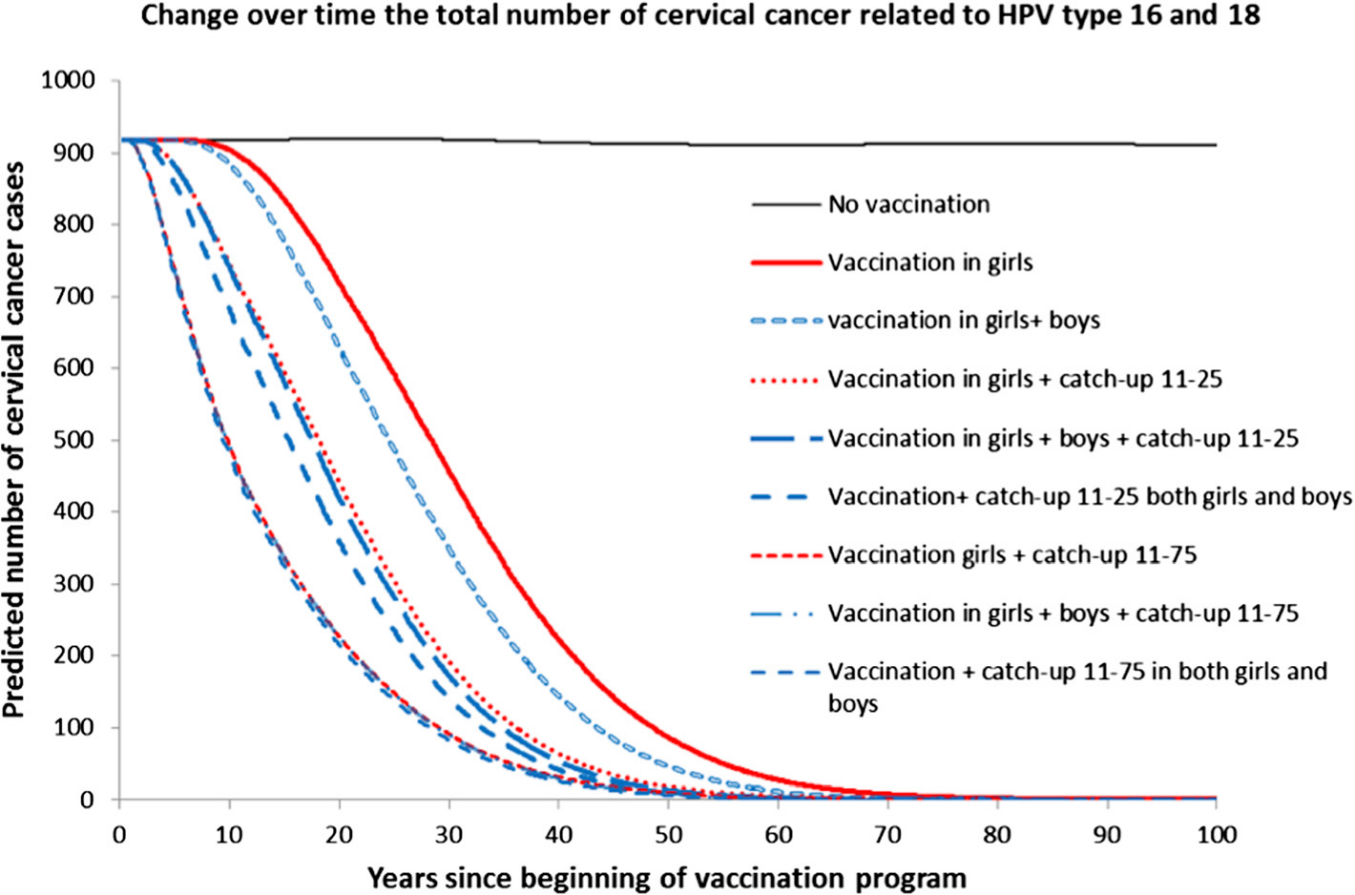
Change over time the total number of cervical cancer related to HPV type 16 and 18. The number of cervical cancer decreases over time in the strategies to, either adding boy or adding a catch-up vaccination component to the girl vaccination. Adding a catch-up component decreases cervical cancer in earlier stage compared to adding a boy vaccination component

The model shows that vaccinating 10-year-old girls has the potential to reduce the number of cervical cancers due to HPV type 16/18 by 78 % and provides a potential diminution of 31 DALYs per 1000 women (Table 3). Furthermore, the model predicts that these benefits increase when a catch-up vaccination and/or a 10-year-old boy vaccination component are added. The reduction of cancer in adding catch-up vaccination was in earlier stage compared to adding boy vaccination (Fig. 3).

**Table 3:** The effectiveness, total cost, and incremental cost-effectiveness by vaccination strategy against cervical cancer due to HPV types 16 and 18

Number	Options	Total cost per 1000 women	Cancer per 1000 women	Cancer reduction (%)	DALY averted per 1000 women	Cost-effective ratio (cancer reduction)	Cost-effective ratio (DALY averted)	ICER (cancer reduction)	ICER (DALY averted)
1.	No vaccination with current screening	4497	3.4	-	-	-	-	-	-
2.	10 years old girls	21,599	0.7	77.9	31.1	8000	695	6334	550
3.	10 years old girls + catch-up girls aged 11–25 years old	27,807	0.4	86.8	35.1	9269	792	20,693	1559
4.	10 years old girls + boys	38,030	0.6	81.3	32.6	13,582	1167	D	D
5.	10 years old girls + catch-up girls aged 11–75 years old	39,059	0.3	91.4	37.0	12,600	1056	112,520	5840
6.	10 years old girls + boys + catch-up girls aged 11–25 years old	44,263	0.4	87.4	35.3	14,754	1254	D	D
7.	10 years old girls + boys + catch-up girls and boys aged 11–25 years old	50,210	0.4	88.7	35.9	16,737	1399	D	D
8.	10 years old girls + boys + catch-up girls aged 11–75 years old	55,520	0.3	91.4	37.0	17,910	1501	D	D
9.	10 years old girls + boys + catch-up girls and boys aged 11–75 years old	72,723	0.3	91.8	37.2	23,459	1955	D	168,320

Note: The cost-effectiveness ratio was a comparison of an option and the baseline scenarios (no vaccination with current screening). The incremental cost of effectiveness ratio expressed as cancer prevented or DALY averted is listed in order of increasing cost. In non-dominant strategy, the ICER was calculated by devising different cost to different effectiveness. ICERs is comparing the option to the next best alternative option. The D refers to strong dominance, which is expressed as higher cost, but lower effectiveness than alternative options. The currency is 2013 international dollars, using purchasing power parity

The GDP per capita in 2013 was about 4822 international dollars [30]

In terms of cost, the baseline strategy (no vaccination with 5.2 % coverage of conventional cytology screening) is the cheapest option, followed by a 10-year-old girl vaccination programme and a catch-up vaccination component for 11–25 year-old females, respectively. In terms of ICER per cancer prevented, the female vaccination option is cost-effective, with an ICER of I$ 6334 per cancer prevented, which is between 1 and 3 GDP per capita. Adding a catch-up vaccination component for females aged 11–25 years old, or a boy vaccination component to the girl vaccination programme does not appear to be cost-effective since their ICERs are higher than 3 GDP per capita. Other strategies are dominated by adding catch-up component for 11–75 year-old females. In terms of ICER per DALY averted, compared to the baseline, the model shows that the girl vaccination option is very cost-effective. Adding a catch-up vaccination component for 11–25 year-old females is very cost-effective compared to the girl vaccination option alone. Moreover, extending the age limit of the catch-up component up to 75 years is also cost-effective. In contrast, adding a boy vaccination component to the girl vaccination option alone, or along with a catch-up component, does not appear to be cost-effective (Table 3).

Table 4 shows the model output results when varying the upper ages for the catch-up component in females. The addition of a catch-up component remains very cost-effective until an upper age of 40 years.

**Table 4:** The cost-effectiveness of catch-up vaccination by upper age limit

Number	Options	Total cost per 1000 women	DALY averted per 1000 women	Cost-effective ratio (DALY averted)	ICER (DALY averted)
1.	No vaccination with current screening	4497	-	-	-
2.	Catch-up 11–18	24,680	34.1	591.9	592
3.	Catch-up 11–20	25,548	34.4	611.9	2373
4.	Catch-up 11–22	26,446	34.7	632.5	3476
5.	Catch-up 11–25	27,807	35.1	664.1	3420
6.	Catch-up 11–30	29,983	35.7	713.9	3880
7.	Catch-up 11–35	31,919	36.2	757.5	3882
8.	Catch-up 11–40	33,584	36.5	796.9	4662
9.	Catch-up 11–45	34,991	36.7	830.9	7012
10.	Catch-up 11–50	36,159	36.9	858.0	8408
11.	Catch-up 11–55	37,109	36.9	883.8	D
12.	Catch-up 11–60	37,857	37	901.6	15,750
13.	Catch-up 11–65	38,418	37	916.8	D
14.	Catch-up 11–70	38,812	37	927.4	D
15.	Catch-up 11–75	39,059	37	934.1	D

Note: The cost-effectiveness ratio was a comparison of an option and the baseline scenarios (no vaccination with current screening). The incremental cost of the effectiveness ratio expressed as DALY averted is listed in order of increasing cost. In non-dominant strategies, the ICER was calculated by dividing different costs by different effectiveness. The intervention was compared to the next more effective and more costly option. The D refers to strong dominance, which is expressed as a higher cost and a lower effectiveness compared to the alternative options. The currency is 2013 international dollars, using purchasing power parity

The GDP per capita in 2013 was about 4822 international dollars [30]

### Sensitivity analyses

Sensitivity analyses show that the parameters that have the greatest impact on ICERs per DALY averted are vaccination coverage, cost of vaccine, discount rate, incidence of cervical cancer, duration of vaccine protection and of natural immunity, and efficacy of the vaccine. The cost of cancer treatment and the disability weighting had no impact on ICERs.

The girl vaccination program is robust to changes in vaccination costs. But the ICER for a catch-up component for 11–25 year olds is higher than one GDP per capita when the cost of the vaccine is I$ 50 or higher per dose. Meanwhile, the model predicts that adding a catch-up vaccination component for females aged 11–75 years old becomes very cost-effective compared to a catch-up for females aged 11–25 years old in the following situations: 1) vaccination coverage is 50 % or lower, 2) vaccine effectiveness is 30 % or lower, 3) the incidence of cervical cancer increases to 40 %, 4) the duration of natural immunity and of vaccine protection is no longer than 10 years, 5) DALYs are not discounted, and 6) the discount rate is 5 % for DALYs and 6 % for the costs.

Moreover, the predicted cost-effectiveness was influenced by the time horizon. If the time horizon is reduced to 30 years then the strategy of adding a boy vaccination and catch-up component for both females and males aged 11–25 years old is predicted to become cost-effective (Additional file 1).

The model predicts that the cost-effectiveness of HPV vaccination is not affected by different initial ages of girl vaccination. When taking into account all cervical cancer cases, we find that the number of cancers due to other high-risk HPV increases by about 2 % from the baseline. However, this does not change the cost-effectiveness results (Additional file 1).

To evaluate the generalizability of results, we calibrated the model to different populations in terms of population size and demographic structure, i.e. Vientiane province. The results were robust to these changes (Additional file 1).

## Discussion

Our model suggests that vaccinating 10-year-old girls is very cost-effective even if the vaccine is expensive (I$ 100 per dose). Adding a boy vaccination component produces little additional benefit, with a further reduction in cervical cancers of just of 3.4 %. As a result, adding this component is less effective than a girl vaccination along with a catch-up vaccination component for 11–25 year-old women, which results in a further reduction of 8.9 % in the number of cancers and an additional diminution of 5 DALYs per 1000 women. This catch-up vaccination component becomes the most attractive strategy with a cost per DALY below one GDP per capita. This result is similar to that found in a previous review [17].

Moreover, the model predicts that adding a catch-up component for females aged 11–25 years old is more attractive than adding a catch-up component for an older age group, if GDP per capita is considered. This age group was also found to be cost-effective in the studies of Elbasha and colleagues [18] and Dasbach colleagues [19]. However, to provide more comprehensive information regarding the appropriate maximum limit age in the catch-up component, which was not reported in previous studies [19], we compared further maximum ages, from 18 to 75, using 5-year intervals. Our study found that a catch-up component for women up to 40 years old was the most attractive option, costing less than one GDP per capita per DALY averted. Several reasons can be proposed. First, the ideal age for a catch-up component might depend on sexual behavior. Second, the prevalence of HPV infection in our model simultaneously decreases after 40 years of age. Finally, one should consider that the incidence of cervical cancer increases after 40 years of age. However, our results should be interpreted cautiously because our model was not calibrated to age-specific HPV prevalence, although the trend of HPV prevalence in Lao PDR seems to be similar to that found worldwide [20]. Also, a clinical trial showed that the vaccine was safe and that it conferred a high-level of immunogenicity in women up to the age of 45 years [21].

Nevertheless, in the case of a higher burden of the disease, or waning of natural immunity, or a suboptimal protection from the vaccine in terms of duration, effectiveness, or vaccination coverage, implementing a catch-up component for females aged 11–75 years old is the most attractive option. This parameter’s influence was also reported by Jit and colleagues [6] and by Van de Velde and colleagues [22]. Indeed, the effectiveness of the vaccination increases when 1) the incidence of cervical cancer is high or 2) the natural immunity wanes; contrarily, this effectiveness decreases in other cases. However, both situations lead to the same conclusion, which is that it is more efficient to vaccinate larger female populations. The lower effectiveness of vaccines might be true in developing countries due to the fact that the HPV vaccine also requires an appropriate maintenance and delivery process [23]. In Lao PDR, a low optimal efficacy of vaccination was reported for hepatitis B vaccine, at only around 65 %. The rate is even lower still in rural areas [24]. Moreover, a low vaccination coverage might be found in rural settings where fewer girls have been found to attend school regularly [8].

Furthermore, it is more cost-effective to include a boy vaccination component in addition to the catch-up component to the girl vaccination program if the time covered by the simulation is shorter, 30 years for instance. This reflects the insufficient level of vaccination protection in the population in the early stages of implementation.

Our study had some limitations. First, our model did not take into account any cross-protection provided by the vaccine to other HPV-related diseases, such as warts and other cancers. This might underestimate the total DALYs averted related to all HPV types. However, this might not significantly bias our conclusion because of the slight benefits provided by this cross-protection [25]. Second, we have ignored some items related to screening and treatment. These include the cost of specimen delivery and the cost of treatment complications. This might lead to an underestimation of the total cost per person. However, according to Goldhaber-Fiebert and Goldie [26], these cost components are small relative to the cost of screening and treatment. Third, it is likely that newer vaccines, active against multiple HPV types, will provide even greater levels of protection [27]. It has also been reported that two doses of HPV vaccine are equally effective in producing immunity as three doses [28]. This might further reduce the cost, and subsequently increase the cost-effectiveness of the vaccination, as demonstrated in a cost-effectiveness study in the UK [29], As our study did not take into account these aspects, future studies might be necessary to investigate these factors for Lao PDR. Finally, DALYs as the outcome of interest might be less interpretable compared to Quality-Adjusted Life Years (QALYs), because they do not reflect as precisely sociocultural considerations regarding health states. Moreover, the discounting rate used for DALY is controversial because it leads to age discrimination. However, there is no valid instrument to measure utilities in the Lao population. A standard gamble method was tried out to produce utilities scores for a range of health problems in a sample of Lao students, but could not come out with sensible results (Daniel Reinharz, personal communication). That is why DALYs were used as recommended by WHO [15].

Finally, one should stress that the study does not reflect the financial affordability of the health care system in Lao PDR. The threshold ratio used to measure the cost-effectiveness is the GDP per capita, which is controversial. Moreover, the limited resources in the country lead to strong competition among interventions in health care programmes. Accurate data on the burden of this disease in Lao PDR would provide important information for decision makers.

## Conclusions

Vaccinating 10-year-old girls with a catch-up program component for 11–25 year-old women is the most attractive option for Lao PDR in 100 years. The girl HPV vaccination, in combination with a catch-up component, should at least be considered for nationwide implementation in Lao PDR.

## Additional file

Additional file 1: Detailed methods and results for economic evaluation. (DOCX 6847 kb)

## Abbreviations

CIN: Cervical intraepithelial neoplasia
DALYs: Disability-adjusted life years
GAVI: Global alliance for vaccines and immunization
GDP: Gross domestic product
HPV: Human papillomaviruses
ICER: Incremental cost-effectiveness ratio
Lao PDR: Lao People’s democratic republic
RAS: Realistic age structured
