# Supplementary material for: The economic evaluation of human papillomavirus vaccination strategies against cervical cancer in women in Lao PDR: a mathematical modelling approach
Source: BMC Health Serv Res. 2016 Aug 22;16(1):418. doi: 10.1186/s12913-016-1662-5 (PMC4994168; doi:10.1186/s12913-016-1662-5)
Supplement: Additional file 1: — Detailed methods and results for economic evaluation. (DOCX 6847 kb) [file 12913_2016_1662_MOESM1_ESM.docx]

**Appendix**

# Methodology

A simulation of the cost-effectiveness of cervical cancer prevention strategies was conducted. Two outcomes were considered as denominators: 1) the number of cervical cancers related to HPV types 16 and 18, and 2) disability adjusted life years (DALYs) related to HPV types 16 and 18. The numerator consisted of the direct cost to the public healthcare system of each strategy. This economic evaluation study complied with the recommendations of the WHO for cost-effectiveness analyses.[^1^](#_ENREF_1)

## Simulation overview

Using a mathematical approach, a compartmental dynamic model of the natural history of HPV infection and cervical cancer was constructed and calibrated to reflect the Vientiane Capital province, Lao PDR population in terms of age and sex distribution,[^2^](#_ENREF_2) as well as the age-specific incidence and mortality rates related to cervical cancer in 2014.[^3^](#_ENREF_3) The model consisted of a dynamic cohort population categorized in one-year age groups. The model considered the occurrence of HPV infection and its progression to precancerous lesions and invasive cervical cancer,[^4^](#_ENREF_4) according to the probabilities of administrating a context-appropriate treatment for cervical precancerous and invasive cancers. Events defined in the model (such as cervical intraepithelial neoplasia (CIN), cervical cancer, death) were probabilistically monthly imputed to the virtual population over the time course of the simulation. The parameters were retrieved from the literature. The vaccination strategies consisted of including a 10-year-old boy vaccination program and/or a catch-up vaccination element for different age groups of the population.

The virtual population was processed over a period of 100 years. This period of time was used to capture the long-term impact of HPV vaccination.[^5^](#_ENREF_5) Incremental cost-effectiveness ratios were computed based on the simulation results. Sensitivity analyses were performed on a specific set of parameters expected to be the most influential on the outcomes.[^6^](#_ENREF_6)

## Virtual population

The baseline virtual population (at year 1) consisted of the entire population of women with characteristics similar to the 2014 Vientiane Capital population in terms of age and sex distribution[^2^](#_ENREF_2) and age-specific incidence and mortality rates of cervical cancer (in one-year intervals).[^3^](#_ENREF_3) The Vientiane Capital population was used in the model instead of the whole country due to the fact that the population of the country is predominantly rural,[^7^](#_ENREF_7) and the ethnic mix of the population[^8^](#_ENREF_8) is likely to be very different in each of the provinces; subsequently the vaccination uptake might be different.

## Scenarios

The scenarios consisted of 1) a baseline which referred to the situation of no-vaccination and a prevalence of cytology-based screening of 5.2%,[^3^](#_ENREF_3) reflecting the situation before the implementation of a preadolescent girl vaccination programme in Vientiane Capital, 2) a 10 year-old girl vaccination programme, 3) a girl and boy vaccination programme, 4) a 10 year-old girl vaccination programme with a catch-up component for females aged 11-25 years, 5) a girl and boy vaccination programme with a catch-up component for females aged 11-25 years, 6) a girl and boy vaccination programme with a catch-up component for females and males aged 11-25 years, 7) a girl vaccination programme with a catch-up component for females aged 11-75 years, 8) a girl and boy vaccination programme with a catch-up component for females aged 11-75 years, and 9) a girl and boy vaccination programme with a catch-up component for females and males aged 11-75 years.

The 10-year-old girl vaccination programme was chosen because the vaccine ensures maximum benefit if administered to girls before they become sexually active and also because this age group is more easily reachable through schools. The first age group for the catch-up vaccination component was selected because the 11-25 year old age group represents the age of students who are reachable through school and university-based interventions. The 11-75 year old age group represents the population at risk of HPV infection in our model.

## Model structure

Inspired by previous economic models of HPV vaccination,[^9-11^](#_ENREF_9) a dynamic transmission and compartment population-based model was created to reflect the expected effect of HPV vaccination programs, both in females and males. Susceptible girls and boys were considered to be at risk of infection based on estimated infection rates between partners. For both males and females, the model considered if the HPV genotype was 16, 18, or other high-risk types, or if the genotypes were low risk.

The model considers that among infected women, some lesions regress thanks to natural immunity against a specific HPV type, but these women remain susceptible to infection by other HPV types. An infection might also persist, and may then progress to cervical intraepithelial neoplasia (low-grade CIN “CIN 1” or high-grade CIN "CIN 2/3”, according to Richart’s modified classification).[^12^](#_ENREF_12) A low-grade CIN might regress to either immunity state or infection state,[^13-16^](#_ENREF_13) or progress to a high-grade CIN. In the case of a high-grade CIN, the lesion might regress to immunity state, infection state or low-grade CIN, or might progress and become an invasive cervical cancer (local, regional and distant progression).[^17^](#_ENREF_17)^,^ [^18^](#_ENREF_18) Distant/metastatic cancer can only progress towards death. Additionally, infected women may die of a cause other than cervical cancer. Women diagnosed with precancerous lesions will be treated by either the loop electrosurgical excision procedure (LEEP) or a hysterectomy. Women with invasive cervical cancer might be symptomatically detected. Diagnosed invasive cervical cancer is treated accordingly, with a defined probability of recovery or treatment failure or death due to treatment complications (Figure 1).

In males, the infection might persist or regress conferring on them a natural immunity against a defined HPV genotype. The consequences of HPV infection in males, such as warts, were not included in the model because we were only interested in the impact of HPV vaccination on cervical cancer in women. Males could die from general causes.

The model assumed that vaccinated people who entered into the vaccine protection compartment remained susceptible for HPV genotypes not covered by the vaccine; consequently, they had a certain probability of being infected with HPV and getting an invasive cancer. Vaccinated people were susceptible to infection with HPV types 16 or 18 depending on assumptions made regarding the waning of vaccine immunity.

Lao experts validated the model in order to ensure that it realistically reflected the possible outcomes of routine screening and treatment of patients in the Vientiane Capital context.

## Parameters

The infection rate depended on the age-specific number of new sexual partners per month, the HPV genotype-specific transmissibility, and the age-specific HPV prevalence in the opposite sex. To simplify the model, we considered all members of the population as heterosexuals. With each sexual partner, the HPV infection is probabilistically transmitted, depending on genotype-specific transmission probabilities and age-specific HPV prevalence in the opposite sex population. A sexual relationship matrix group was constructed. The matrix consisted of the monthly age-specific probability of having new sexual partners. Each age group had a probability of having sexual intercourse with someone of the same or a different age group of 0.6 and 0.4 respectively, based on a previous national survey.[^19^](#_ENREF_19) The initial age of sexual intercourse is 15 years old or over in both females and males, according to the most recent survey performed in Vientiane Capital province.[^20^](#_ENREF_20) Due to unknown parameters of the number of new sexual partners in Lao PDR, data from the UK[^10^](#_ENREF_10) were used and calibrated to the age-specific incidence of cervical cancer in Lao PDR. The transmissibility of each HPV type was calibrated to take into account the proportion of genotype-specific HPV prevalence and the proportion of cervical cancers due to HPV types 16 and 18 (Table 2). The proportion of HPV types 16 and 18 among all-type HPV infections was, based on Thai data,[^21^](#_ENREF_21) assumed to be 45-50%. These infections may reasonably be assumed to be responsible for approximately 75% of the total incidence of invasive cervical cancer.[^22^](#_ENREF_22)

Monthly transition probabilities from one lesion state to another and regression rates were taken from Kim colleagues.[^23^](#_ENREF_23) For instance, the age-specific monthly probability that a HPV type 16 infection progresses to a low-grade CIN is 0.0047-0.0085, while the rate of transition from low to high-grade CIN is 0.0001-0.0039. The annual rate of detecting an invasive cervical cancer via symptoms is 0.19, 0.6 and 0.9 for local, regional and distant cervical cancers, respectively (Table 3).

A true positive result of cervical cytology was defined as a high-grade CIN. We assumed that 55% of these cases would receive the complete treatment regimen, considering a 15% loss to follow-up over the three expected visits. The first visit is for screening, the second for receiving the result and making an appointment in the event of a positive diagnosis. The third visit is for a colposcopy with direct biopsy. The proportion of treatment with either LEEP or cryotherapy was based on expert opinion. The rate of remission was retrieved from the literature.[^24^](#_ENREF_24)^,^ [^25^](#_ENREF_25) The expert panel consisted of two gynaecologists whose practice focused on cervical cancer in Lao PDR.

Among diagnosed patients the proportion of women receiving cancer treatment, and the stage-specific five-year survival rates due to cancer treatment complications, were calibrated based on the estimated mortality rates related to cervical cancer according to Globocan, 2012 (Table 3).[^3^](#_ENREF_3)

The sensitivity and specificity of conventional cervical cytology to detect a high-grade CIN or worse were considered to be 59% (range: 29%-82%) and 94% (range: 88%-99%), respectively.[^26^](#_ENREF_26) The model considered that colposcopy with direct biopsy is used to confirm a positive result from a cervical cytology test. The sensitivity and specificity of colposcopy were considered to be 96% (64-99%) and 48% (30-93%), respectively. Biopsy was assumed to have a sensitivity and specificity of 100% (Table 4).

***Precancerous lesions and cancer stage treatment***

Success rates for LEEP and hysterectomy were considered to be 96.7% (90-98%) and 99% (90-100%), respectively.[^25^](#_ENREF_25) The proportion of diagnosed women treated with either LEEP or hysterectomy depended on their age. For women aged 35 years or less, it was considered that 80% (50-100%) would be treated with LEEP and 20% (0-50%) with a hysterectomy. For those over 35 years old, the numbers were reversed: 20% (0-50%) with LEEP and 80% (50-100%) with hysterectomy. The remission rate of stage-specific invasive cervical cancer was calibrated based on the estimated mortality related to cervical cancer in Lao PDR[^3^](#_ENREF_3) (Table 4).

## Model calibration

The population was stratified by gender and age. The model was in the form of a realistic age structured (RAS) model. The equations were numerically solved in Berkeley Madonna version 8.3.18.[^27^](#_ENREF_27) The model was calibrated using maximum likelihood for the age-specific distribution of the 2014 data on the estimated incidence of cervical cancer and mortality related to cervical cancer in Lao PDR. Thai data on the prevalence of HPV infection and the prevalence of low-grade and high-grade CIN were used to guide their age-specific distributions. The demographic distribution followed an exponential distribution using UN data to predict the changing birth and death rates over time for Lao PDR.[^28^](#_ENREF_28) To calibrate the age-specific incidence of cervical cancer, we assumed that only the infection rate was different from the Kim and colleagues model.[^23^](#_ENREF_23) We consequently calculated an infection rate multiplier to calibrate the incidence of cervical cancer according to the Globocan estimates and used under- and over-estimates in sensitivity analyses (Table 5).

The calibration of parameters for the age- and stage-specific mortality rates of cervical cancer was conducted by varying the proportion of women receiving treatment for local, regional and distant cancer, the monthly death rates due to treatment complications, and the age- and stage-specific remission rates. The true proportion of women receiving treatment in Lao PDR is unknown; we therefore estimated its value according to expert opinion. The best guess of the proportion of women receiving treatment for a local, regional or distant cancer was 100%, 80% and 70%, respectively (Table 3).

## Costs

One should stress the fact that no economic evaluation of health interventions using mathematical models has ever been done previously in Lao PDR. This section refers therefore to a component of the model that required some approximations, as structures supporting the healthcare system to provide the required information for conducting economic evaluations do not currently exist. We recognise that this is a limitation of this work, but considered that by including estimates for this component we would open doors to the realisation of further studies on value for money spent in the Lao PDR healthcare sector.

The perspective considered was essentially the perspective of the public healthcare system. Only direct medical costs and the programmatic cost of vaccination implementation were considered.

***Items***

Items were related to the consumption of medical resources for the diagnosis and treatment of cervical cancer and HPV (screening and laboratory facilities, diagnostic tests, hospitalisations, and treatment), as well as the vaccination cost (programmatic cost). A preliminary list of items was built with the help of gynaecologists and pathologists working in Lao PDR. These items consisted of:

1. Screening-related items: these included support items, medical administration, and labour costs. Support item costs included electricity, water, transportation, supplies and other office materials, and support staff. Medical administration included training support and medical equipment. Labour costs included the time spent by the gynaecologist and the nurse for screening activities. Cytology requires three visits. The first visit is for screening, the second for receiving the result and making an appointment in the event of a positive diagnosis. The third is for a colposcopy with direct biopsy.
2. Laboratory related items: these items were listed according to a pathologist’s advice. Cervical cytology and histology examinations included administration, consumables, and labour costs. Consumable items for cervical cytology included cover glasses, malinol, Gill’s hematoxylin, OG-6, EA-50, masks, xylene, ethanol, and slides. For histology exams, the materials included formaline, hematocyline, eosine, Paraffin, cassette, cyline, absolute alcohol, acetone and malinone. In Vientiane Capital province, four pathology technicians work together and can prepare a total of 50 smear slides for conventional cervical cytology per day. They can also prepare ten histology slides per day. A pathologist needs between 20-35 minutes for cytology and histology examinations. Other materials used for a cytology examination could not be identified due to lack of information (Table 7).
3. Medication and surgery: items required for precancerous lesion treatment included support activities, drugs, and equipment and labour costs. LEEP requires one day of hospitalisation and a simple hysterectomy requires seven days in hospital.
4. Vaccination: this included both the vaccine cost and the programmatic cost, which included micro-planning, training, social mobilisation, procurement, logistics, service delivery, supervision, and waste management.
5. The programmatic cost of screening included quality control, training, administration, and recruitment costs.

***Quantification***

There are no national guidelines for cervical cancer control in Lao PDR and no data bank where information can be retrieved. Quantities were therefore estimated based on expert opinion.

1. Time spent for screening is estimated to be on average about 20 minutes for cervical cytology. Time spent on cervical cytology and histology interpretation is estimated to be between 20 and 35 minutes per case, respectively.
2. The number of visits considered necessary for screening and treatment is three.
3. Only consumable items used in the cytology and histology laboratory were considered. In Vientiane Capital province, four pathology technicians work together and take one day to prepare 50 to 80 smear slides for conventional and liquid-based cervical cytology, respectively. They can also prepare ten histology slides per day. A pathologist needs 20 to 35 minutes to examine each cytology and histology case, respectively.
4. Other quantities were approximated, for example hospitalisation and surgery.

***Item pricing***

Unit prices are reported in the value of 2013 international dollars (I$), using purchasing power parity (PPP). According to the WHO, a PPP exchange rate is the number of units of a country’s currency required to buy the same amount of goods and services in the domestic market as can be bought with one US dollar in the United States. International dollars are, therefore, a hypothetical currency allowing comparisons and integration of costs between countries.[^29^](#_ENREF_29) In the present study unit prices are given in 2013 international dollars, using the PPP exchange rate of one international dollar (I$) = 2 694.27 Lao kip).[^29^](#_ENREF_29)

Cost per service was calculated by multiplying the price per unit by the amount of units consumed. Where possible, unit prices were based on data from Lao PDR. A Lao PDR hospital unit price list is available, with prices estimated through a costing survey performed at the departments of gynaeco-obstretics of two reference hospitals in Vientiane, Mahosot and Setthathirath hospitals, during 2013-2014 (personal communication, 2014). The survey applied a step-down allocation method to estimate an average unit price per visit and per hospitalisation. Capital costs were not considered due to the difficulty to make an estimation of their real value. Unit prices for missing items were retrieved from the literature deemed relevant for the Lao situation. The Lao expert committee validated the accuracy of the valuation procedure.

The costs of administration and labour in a screening facility are 14.48 and 3.39 I$, respectively (Table 6). For the unit price of the LEEP we used the average cost of one-day’s hospitalisation in a gynaeco-obstetric ward. The unit price of a simple hysterectomy was considered to be the same as the average unit price of any surgical operation. Complications with cryotherapy, LEEP and hysterectomy are rare; therefore they were not considered (Table 8).

The unit price of invasive cervical cancer treatment was retrieved from a study done in 72 Global Alliance for Vaccines and Immunization (GAVI)-eligible countries.[^30^](#_ENREF_30) It included the costs of treatment for localised, regional and distant cervical cancers (Table 8).

***Vaccination cost***

The cost of delivering HPV vaccines consisted of the price of the vaccine and the programmatic cost of vaccination delivery. The programmatic cost of a three-dose HPV vaccine per girl was retrieved from a pilot project on HPV vaccination in 5^th^ grade girls in Vientiane Capital in 2014. The programmatic cost included micro-planning, training, social mobilisation, procurement, logistics, service delivery, supervision, and waste management. The vaccine price per dose was based on the purchasing cost from GAVI (4.5 US dollars per dose)[^31^](#_ENREF_31) (Table 4).

## Simulation analyses

The simulation process was run deterministically over a 100-year time span to capture the short and long term benefits of vaccination. For each option, the output consisted of the cumulative number of cervical cancers per 1 000 women, the DALYs per 1 000 women, and the cost of screening and treatment per 1 000 women. DALYs were calculated based on the WHO table without age weighting. The disability weighting for cancer treatment was retrieved from the current literature.[^32^](#_ENREF_32) For each strategy, a cost-effectiveness ratio was calculated using the reduction in numbers of cervical cancers and DALYs averted as denominators. In the case of a non-dominant situation, or strong or extended dominance, the incremental cost-effectiveness ratio (ICER) was calculated, expressing the incremental cost per unit of health benefit gained compared to the alternative intervention, which is generally the next option characterised by a higher cost and a higher effectiveness. Two types of dominance can be defined: the strong dominant type in which an option is more effective and less costly than its alternative, and the extended dominant type, also known as weak dominant, where strategies with a higher ICER are ruled out.[^33^](#_ENREF_33)

All costs and DALYs were discounted at a rate of 3% in base case simulations to convert future costs and life expectancies and duration of disability to their present value.[^1^](#_ENREF_1) However, other discount rates of 0% to 5% for DALYs and 6% for costs were also explored.

The results were interpreted taking into account the recommendations of the UN Commission on Macroeconomics and Health which proposes classifying cost-effectiveness studies into three categories: 1) highly cost-effective (ICER < 1 GDP per capita; 2) cost-effective (ICER between 1-3 times the GDP per capita); and 3) not cost-effective (ICER > 3 times the GDP per capita).[^34^](#_ENREF_34) The GDP per capita in Lao PDR in 2013 was about 4 822 international dollars using the PPP exchange rate.[^35^](#_ENREF_35)

## Sensitivity analyses

One-way sensitivity analyses were conducted on parameters using their lower and upper bound values retrieved from the literature to identify the parameters that might significantly influence the incremental cost-effectiveness ratio per DALY averted. One-way sensitivity analyses were performed by varying the values of the incidence of cervical cancer, vaccination coverage, vaccine efficacy, duration of vaccine protection, duration of natural immunity, cost of vaccine per dose, cost of cancer treatment, and discount rate.

Other sensitivity analyses were conducted to explore various factors, such as the initial age of vaccination in girls (11, 12, and 13 years old), the effect of cervical cancers due to non-HPV types 16/18, the effect of ten consecutive cohorts vaccination only, a time horizon of 30 and 50 years, and a programme consisting of vaccinating boys only.

## Model validation process

The model was able to reproduce the 2014 Vientiane Capital expected values regarding demographic data, both for the female and the male populations. However, the number of individuals was high for 10 to 25 year-old individuals compared to expected values, while it was low for 25-35 year-old individuals. The model reproduced results that were consistent with the incidence of cervical cancer and its mortality due to any high-risk HPV type according to the estimates of Globocan 2012 (Figure 2). The proportion of cervical cancers related to HPV types 16 and 18 was about 75%. The calibrated infection rate was not different to that reported in the literature.

**Figure 1: Model structure for the natural history of human papillomavirus infection and cervical cancer**


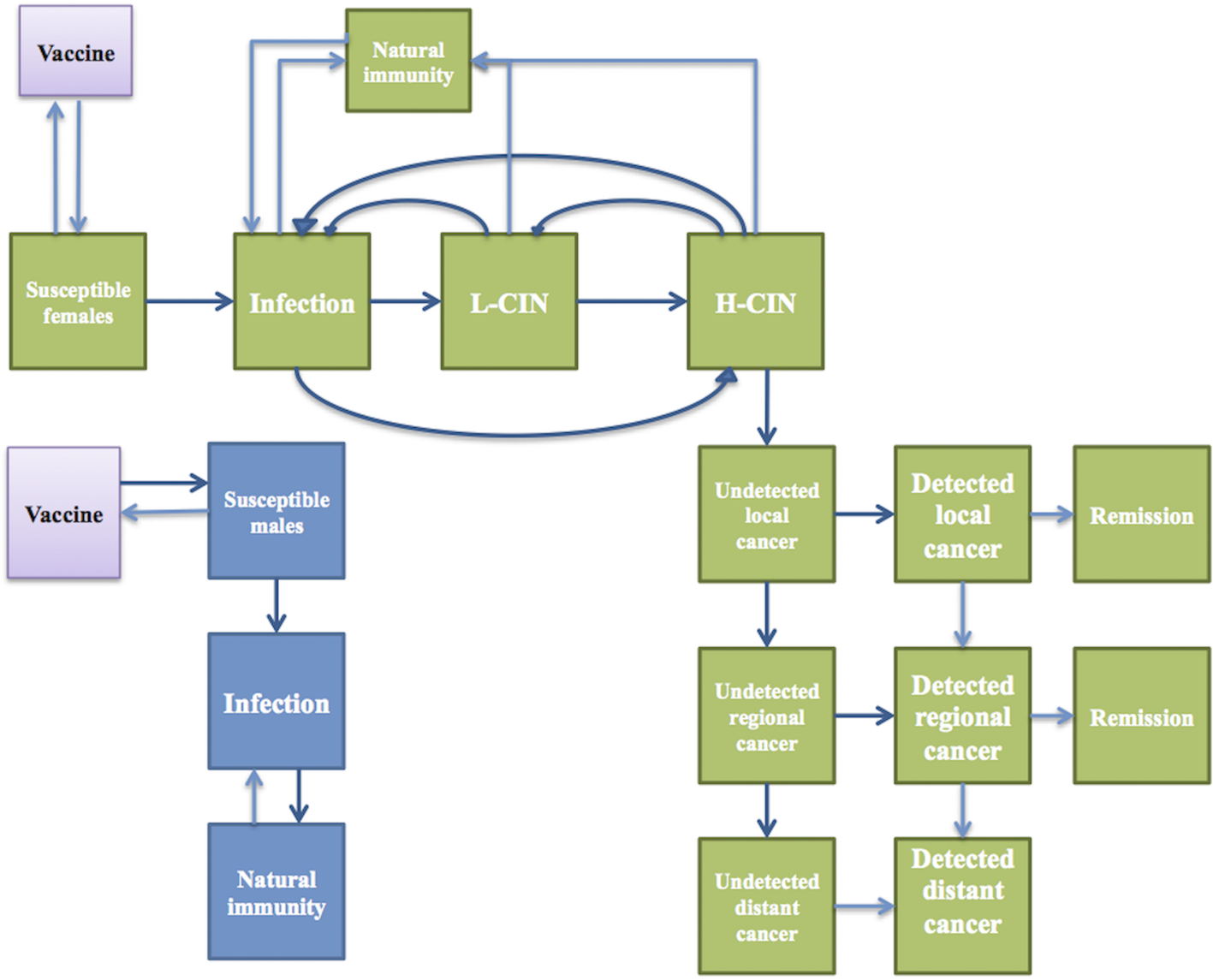


**Overview of the ordinary differential equations**

**State transition equations**

***Female model (1)***

| ****** |
| --- |

$${S'}_{k,0}=\mu P+\epsilon S_{(k-1),0}+\epsilon S_{(k-1),0}+\omega_{k}{RW}_{k,g,0}-{[\lambda}_{k,g}+{\theta_{k}+(\nu_{k}+\rho}_{k}{)\tau}_{k}+\epsilon]S_{k,0}$$

${S'}_{k,1}={\epsilon S_{(k-1),1}+[(\nu_{k}+\rho}_{k}{)\tau}_{k}]S_{k,0}+ \omega_{k}{RW}_{k,g,1}-{(\lambda}_{k,g}+\varphi+\theta_{k}+\epsilon)S_{k,1}$where vaccinated people remains susceptible for other HPV types rather than type 16/18

$${S'}_{k,2}={\epsilon S}_{(k-1),1}+\varphi S_{k,g,1}+\omega_{k}{RW}_{k,g,2}-{(\lambda}_{k,g}+\theta_{k}+\epsilon){SV}_{k,2}$$

$${I'}_{k,g,0}= \epsilon I_{\left( k-1 \right),g,0}+\lambda_{k,g}[S_{k,0}+(1-{x_{g})RW}_{k,g,0}]+ \alpha_{k,g}{CL}_{k,g,0}+\psi_{k,g}{CH}_{k,g,0}-[\gamma_{k,g}+\eta_{k,g}+\partial_{k,g}{+(\nu_{k}+\rho}_{k}{)\tau}_{k}+\theta_{k}+\epsilon]I_{k,g,0}$$

$${I'}_{k,g,1}= \epsilon I_{\left( k-1 \right),g,1}{+ [(\nu_{k}+\rho}_{k}{)\tau}_{k}]I_{k,g,0}+\lambda_{k,g}[S_{k,1}+(1-{x_{g})RW}_{k,g,1}]+ \alpha_{k,g}{CL}_{k,g,1}+\psi_{k,g}{CH}_{k,g,1}-(\gamma_{k,g}+\eta_{k,g}+\partial_{k,g}+\varphi+\theta_{k}+\epsilon)I_{k,g,1}$$

$${I'}_{k,g,2}= \epsilon I_{\left( k-1 \right),g,2}+\varphi I_{k,g,1} +\lambda_{k,g}[S_{k,2}+(1-{x_{g})RW}_{k,g,2}]+ \alpha_{k,g}{CL}_{k,g,2}+\psi_{k,g}{CH}_{k,g,2}-(\gamma_{k,g}+\eta_{k,g}+\partial_{k,g}+\theta_{k}+\epsilon)I_{k,g,2}$$

$${CL'}_{k,g,0}= {\epsilon{CL}_{\left( k-1 \right),g,0}+\eta_{k,g}I}_{k,g,0}+{\varpi_{k,g}CH}_{k,g,0}+(1-ê_{k})T{CL}_{k,g,0}-[\pi_{k,g}+\alpha_{k,g}+\delta_{k,g}{+(\nu_{k}+\rho}_{k}{)\tau}_{k}+æ_{k}+\theta_{k}+\epsilon]{CL}_{k,g,0}$$

$${CL'}_{k,g,1}= {\epsilon{CL}_{\left( k-1 \right),g,1}{+ [(\nu_{k}+\rho}_{k}{)\tau}_{k}]{CL}_{k,g,0}+\eta_{k,g}I}_{k,g,1}+{\varpi_{k,g}CH}_{k,g,1}+(1-ê_{k})T{CL}_{k,g,1}-(\pi_{k,g}+\alpha_{k,g}+\delta_{k,g}+æ_{k}+\varphi+\theta_{k}+\epsilon){CL}_{k,g,1}$$

$${CL'}_{k,g,2}= {\epsilon{CL}_{\left( k-1 \right),g,2}+\varphi{CL}_{k,g,1}+\eta_{k,g}I}_{k,g,2}+{\varpi_{k,g}CH}_{k,g,2}+(1-ê_{k})T{CL}_{k,g,2}-(\pi_{k,g}+\alpha_{k,g}+\delta_{k,g}+æ_{k}+\theta_{k}+\epsilon){CL}_{k,g,2}$$

$${CH'}_{k,g,0}=\epsilon{CH}_{\left( k-1 \right),g,0}+{\partial_{k,g}I}_{k,g,0}+{\pi_{k,g}CL}_{k,g,0}+{(1-\varepsilon}_{k})T{CH}_{k,g,0}-(\mathcal{F}_{k,g}+\beta_{k,g}+\psi_{k,g}+\varpi_{k,g}{+(\nu_{k}+\rho}_{k}{)\tau}_{k}+\phi_{k}+\theta_{k}+\epsilon){CH}_{k,g,0}$$

$${CH'}_{k,g,1}=\epsilon{CH}_{\left( k-1 \right),g,1}{+ [(\nu_{k}+\rho}_{k}{)\tau}_{k}]{CH}_{k,g,0}+{\partial_{k,g}I}_{k,g,i}+{\pi_{k,g}CL}_{k,g,i}+{(1-\varepsilon}_{k})T{CH}_{k,g,i}-(\mathcal{F}_{k,g}+\beta_{k,g}+\psi_{k,g}+\varpi_{k,g}+\phi_{k}+\varphi+\theta_{k}+\epsilon){CH}_{k,g,i}$$

$${CH'}_{k,g,2}=\epsilon{CH}_{\left( k-1 \right),g,2}+\varphi{CH}_{k,g,1}+{\partial_{k,g}I}_{k,g,2}+2+{(1-\varepsilon}_{k})T{CH}_{k,g,2}-(\mathcal{F}_{k,g}+\beta_{k,g}+\psi_{k,g}+\varpi_{k,g}+\phi_{k}+\theta_{k}+\epsilon){CH}_{k,g,2}$$

$${RW'}_{k,g,0}= \epsilon{RW}_{\left( k-1 \right),g,0}+{\gamma_{k,g}I}_{k,g,i}+\delta_{k,g}{CL}_{k,g,i}+{\beta_{k,g}CH}_{k,g,i}+ \varepsilon_{k}T{CH}_{k,g,i}+ ê_{k}T{CL}_{k,g,0}-[(1-{x_{g})\lambda_{k,g}+\omega_{k}+\theta_{k}{+(\nu_{k}+\rho}_{k}{)\tau}_{k}+\epsilon]RW}_{k,g,0}$$

$${RW'}_{k,g,1}= \epsilon{RW}_{\left( k-1 \right),g,1}{+ [(\nu_{k}+\rho}_{k}{)\tau}_{k}]{RW}_{k,g,0}+{\gamma_{k,g}I}_{k,g,1}+\delta_{k,g}{CL}_{k,g,1}+{\beta_{k,g}CH}_{k,g,1}+ \varepsilon_{k}T{CH}_{k,g,1}+ ê_{k}T{CL}_{k,g,1}-(1-{x_{g})\lambda_{k,g}+\omega_{k}+\theta_{k}+\varphi+\epsilon]RW}_{k,g,1}$$

$${RW'}_{k,g,2}= \epsilon{RW}_{\left( k-1 \right),g,2}+\varphi{RW}_{k,g,1}+{\gamma_{k,g}I}_{k,g,2}+\delta_{k,g}{CL}_{k,g,2}+{\beta_{k,g}CH}_{k,g,2}+ \varepsilon_{k}T{CH}_{k,g,2}+ ê_{k}T{CL}_{k,g,2}-[(1-{x_{g})\lambda_{k,g}+\omega_{k}+\theta_{k}+\epsilon]RW}_{k,g,2}$$

$$\nu_{k}=\frac{\epsilon C_{k}}{1-C_{k}}$$

$$\rho_{k}=\frac{\epsilon{COV}_{k}}{1-{COV}_{k}}$$

***Invasive cervical cancer model (2)***

$${LCC'}_{k,g}=\mathcal{F}_{k,g}{CH}_{k,g}-\left( \left( \mathfrak{D+}\phi_{k} \right)\mathcal{g}+h+\theta_{k}+\epsilon\right){LCC}_{k,g}$$

$${RCC'}_{k}=h{LCC}_{k}-\left( \left( \mathbb{Q+}\phi_{k} \right)\Omega\mathcal{+H+}\theta_{k}+\epsilon\right){RCC}_{k}$$

$${DCC'}_{k}\mathcal{=H}{RCC}_{k}-\left( \left( \mathcal{L+}\phi_{k} \right)\mathcal{A+}⋏_{k}+\theta_{k}+\epsilon\right){DCC}_{k}$$

$${LCCd'}_{k,g}=\left( \psi+\phi_{k} \right){\mathcal{g}LCC}_{k,g}-\left( h+\mathcal{m}_{k}+\theta_{k}+\epsilon\right){LCCd}_{k,g}$$

$${RCCd'}_{k}=\left( \mathbb{Q+}\phi_{k} \right){\Omega RCC}_{k}+(1-\mathcal{r}_{k})h{LCCd}_{k}-\left( \mathcal{H+}\mathcal{n}_{k}+\theta_{k}+\epsilon\right){RCCd}_{k}$$

$${DCCd'}_{k}=\left( \mathcal{L+}\phi_{k} \right){\mathcal{A}DCC}_{k}+(1-\mathfrak{y}_{k}){\mathcal{H}RCCd}_{k}-\left( \ni_{k}+\theta_{k}+\epsilon\right){DCCd}_{k}$$

$${RC'}_{k}=\mathcal{r}_{k}h{LCCd}_{k}+\mathfrak{y}_{k}{\mathcal{H}RCCd}_{k}-\left( \theta_{k}+\epsilon\right){RC}_{k}$$

***Male model (3)***

| 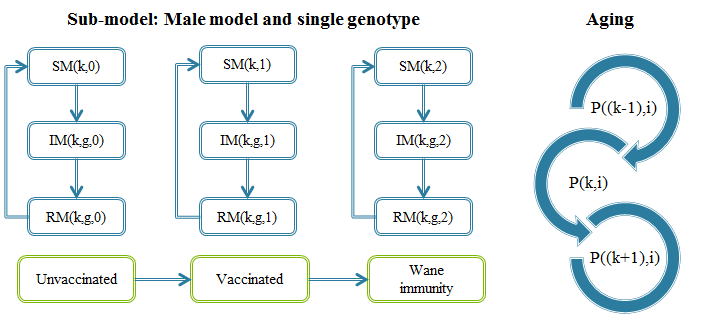 |
| --- |

$${SM'}_{k,0}=\mu\mathcal{o}P+\epsilon{SM}_{(k-1),0}+\omega_{k}{RM}_{k,g,0}-{[\lambda}_{k,g}+{\theta_{k}+(\nu_{k}+\rho}_{k}{)\tau}_{k}+\epsilon]{SM}_{k,0}$$

${SM'}_{k,1}=\epsilon{SM}_{(k-1),1}+[{(\nu_{k}+\rho}_{k}{)\tau}_{k}]{SM}_{k,0} {{+ \omega}_{k}RM}_{k,g,1}- {(\lambda}_{k,g}+\theta_{k}+\varphi+\epsilon){SM}_{k,1}$ where vaccinated people remains susceptible for other HPV types rather than type 16/18

$${SM'}_{k,2}=\epsilon{SM}_{(k-1),2}+\varphi{SM}_{k,1}+{\omega_{k}RM}_{k,g,2}-{(\lambda}_{k,g}+\theta_{k}+\epsilon){SM}_{k,2}$$

$${IM'}_{k,g,0}= \epsilon{RM}_{\left( k-1 \right),g,0}+\lambda_{k,g}[{SM}_{k,0}+(1-{x_{g})RM}_{k,g,0}]-[\gamma_{k,g}{+(\nu_{k}+\rho}_{k}{)\tau}_{k}+\theta_{k}+\epsilon]{IM}_{k,g,0}$$

$${IM'}_{k,g,1}= \epsilon{RM}_{\left( k-1 \right),g,1}+[{(\nu_{k}+\rho}_{k}{)\tau}_{k}]{IM}_{k,g,0}+\lambda_{k,g}[{SM}_{k,1}+\left( 1-{x_{g})RM}_{k,g,1} \right]-[\gamma_{k,g}+\varphi+\theta_{k}+\epsilon]{IM}_{k,g,1}$$

$${IM'}_{k,g,2}=\epsilon{RM}_{\left( k-1 \right),g,2}+\varphi{IM}_{k,g,1}+ \lambda_{k,g}[{SM}_{k,2}+\left( 1-{x_{g})RM}_{k,g,2} \right]-[\gamma_{k,g}-\left( \gamma_{k,g}+\theta_{k}+\epsilon\right){]IM}_{k,g,2}$$

$${RM'}_{k,g,0}=\epsilon{RM}_{\left( k-1 \right),g,0}+ {\gamma_{k,g}IM}_{k,g,0}-[(1-{x_{g})\lambda_{k,g}+\omega_{k}+{\theta_{k}+(\nu_{k}+\rho}_{k}{)\tau}_{k}+\epsilon]RM}_{k,g,0}$$

${RM'}_{k,g,1}=\epsilon{RM}_{\left( k-1 \right),g,1}+{\gamma_{k,g}IM}_{k,g,1}+[{(\nu_{k}+\rho}_{k}{)\tau}_{k}]{RM}_{k,0}-( (1-{x_{g})\lambda_{k,g}+\omega_{k}+\varphi+\theta_{k}+\epsilon)RM}_{k,g,1}$where vaccinated people remains susceptible for other HPV types rather than type 16/18

$${RM'}_{k,g,2}=\epsilon{RM}_{\left( k-1 \right),g,2}+\varphi{RM}_{k,g,1}+{\gamma_{k,g}IM}_{k,g,2}-[(1-{x_{g})\lambda_{k,g}+\omega_{k}+\theta_{k}+\epsilon]RM}_{k,g,2}$$

***Precancerous lesions treatment model (4)***

$${TCL'}_{k,g,0}= {\epsilon{TCL}_{\left( k-1 \right),g,0}+æ_{k}CL}_{k,g,i} -[(\theta_{k}+{(1-\varepsilon}_{k})+\varepsilon_{k}{+(\nu_{k}+\rho}_{k}{)\tau}_{k}+\epsilon]T{CL}_{k,g,0}$$

$${TCL'}_{k,g,1}= {\epsilon{TCL}_{\left( k-1 \right),g,1}+æ_{k}CL}_{k,g,i} +[{(\nu_{k}+\rho}_{k}{)\tau}_{k}]{TCL}_{k,g,0}-[(\theta_{k}+{(1-\varepsilon}_{k})+\varepsilon_{k}+\varphi+\epsilon]T{CL}_{k,g,1}$$

$${TCL'}_{k,g,2}= {{\epsilon{TCL}_{\left( k-1 \right),g,2}+\varphi{TCL}_{k,g,1}+æ}_{k}CL}_{k,g,i} -[(\theta_{k}+{(1-\varepsilon}_{k})+\varepsilon_{k}+\epsilon]T{CL}_{k,g,2}$$

$${TCH'}_{k,g,0}= {\epsilon{TCH}_{\left( k-1 \right),g,0}+\phi_{k}CH}_{k,g,0} -[(\theta_{k}+{(1-\varepsilon}_{k})+\varepsilon_{k}{+(\nu_{k}+\rho}_{k}{)\tau}_{k}+\epsilon]T{CH}_{k,g,0}$$

$${TCH'}_{k,g,1}= {{\epsilon{TCH}_{\left( k-1 \right),g,1}+\phi}_{k}CH}_{k,g,1} -[(\theta_{k}+{(1-\varepsilon}_{k})+\varepsilon_{k}+\varphi+\epsilon]T{CH}_{k,g,1}$$

$${TCH'}_{k,g,2}= {\epsilon{TCH}_{\left( k-1 \right),g,2}+\phi_{k}CH}_{k,g,2} -[(\theta_{k}+{(1-\varepsilon}_{k})+\varepsilon_{k}+\epsilon]T{CH}_{k,g,2}$$

$$\phi_{k}= \varsigma_{k}\varrho(1-los\_final)$$

***Force of infection (5)***

$$\lambda_{k,g}=A\sum_{k=1}^{N} {Lamda}_{k,g}$$

Where A is the adjustment of the total estimated force of infection, and N is the total number of age group and

${Lamda}_{k,g}= \frac{\mathcal{T}_{g}M_{k}{IM}_{k,g}}{{POPM}_{k}}$ for female

$${POPM}_{k}={STT}_{k}+{IM}_{k}+RM+{VM}_{k}$$

And

${Lamda}_{k,g}= \frac{\mathcal{T}_{g}M_{k}{(IM}_{k,g}+{CL}_{k,g}+{CH}_{k,g})}{{POPF}_{k}}$ for male

$${POPF}_{k}=S_{k}+I_{k}+CL+{CH}_{k}+RW+{LCC}_{k}+{RCC}_{k}+{DCCd}_{k}+{LCCd}_{k}+{RCCd}_{k}+{DCCd}_{k}$$

Where M is the contact matrix

$M_{k}= {\mathfrak{s}_{1}MS}_{k}+\frac{{\mathfrak{s}_{2}MS}_{k}}{NG-1}$ where MS is new sexual partnership per month; $\mathfrak{s}_{1}$is the probability of having a sexual partner within the same age group; $\mathfrak{s}_{2}$is the probability of having a sexual partner within a different age group; and NG is the total number of age

***Calibration: maximum likelihood estimation (6)***

${LI}_{k,g}= {ICD}_{k,g}\ln\left( IC \right)-IC$ where ICD is the observed incidence of invasive cervical cancer, and IC is expected incidence of invasive cervical cancer

| **Table 1: Abbreviation of the model structure variables** | |
| --- | --- |
| **Variable** | **Meaning** |
| $S_{k,..}$ | Healthy women (age k, 0 is unvaccinated, 1 is vaccinated and 2 is waned status) at time t |
| $I_{k,g,..}$ | Infection in females (age k, genotype g, 0 is unvaccinated, 1 is vaccinated and 2 is waned status) at time t |
| ${RW}_{k,g,..}$ | Regression of infection or precancerous lesions (age k, genotype g, 0 is unvaccinated, 1 is vaccinated and 2 is waned status) at time t |
| ${CL}_{k,g,..}$ | Low-grade Cervical Intraepithelial Neoplasia (age k, genotype g, 0 is unvaccinated, 1 is vaccinated and 2 is waned status) at time t |
| ${CH}_{k,g,..}$ | High-grade Cervical Intraepithelial Neoplasia (age k, genotype g, 0 is unvaccinated, 1 is vaccinated and 2 is waned status) at time t |
| ${LCC}_{k,g}$ | Undetected local cancer (age k, genotype g) at time t |
| ${RCC}_{k}$ | Undetected regional cancer (age k) at time t |
| ${DCC}_{k}$ | Undetected distant cancer (age k) at time t |
| ${LCCd}_{k,g}$ | Detected local cancer (age k, genotype g) at time t |
| ${RCCd}_{k}$ | Detected regional cancer (age k) at time t |
| ${DCCd}_{k}$ | Detected distant cancer (age k) at time t |
| ${RC}_{k}$ | Recovery from cancer treatment (age k) at time t |
| *P* | Total female population |
| ${SM}_{k,..}$ | Healthy males (age k, 0 is unvaccinated, 1 is vaccinated and 2 is waned status) at time t |
| ${IM}_{k,g,..}$ | Infection in males (age k, genotype g, 0 is unvaccinated, 1 is vaccinated and 2 is waned status) at time t |
| ${RM}_{k,g,..}$ | Recovery with natural immunity in males (age k, genotype g, 0 is unvaccinated, 1 is vaccinated and 2 is waned status) at time t |
| ${TCL}_{k,g,..}$ | Women with low-grade CIN receiving treatment (age k, genotype g, 0 is unvaccinated, 1 is vaccinated and 2 is waned status) at time t |
| ${TCH}_{k,g,..}$ | Women with high-grade CIN receiving treatment (age k, genotype g, 0 is unvaccinated, 1 is vaccinated and 2 is waned status) at time t |
| ${POPF}_{k}$ | Total female population (age k) |
| ${POPM}_{k}$ | Total male population (age k) |
| IW16 | HPV type 16 infected women |
| IW18 | HPV type 18 infected women |
| IW_H | Other high-risk HPV infected women |
| RW16 | Clearing up HPV type 16 infection with natural immunity against HPV type 16 |
| RW18 | Clearing up HPV type 18 infection with natural immunity against HPV type 18 |
| RW_H | Clearing up other high-risk HPV infection with natural immunity against high-risk HPV |
| DG | Death due to other causes |
| DC | Death due to cervical cancer |

| **Table 2: Abbreviation of model structure parameters** | |
| --- | --- |
| **Parameters** | **Meaning** |
| $\epsilon$ | Aging rate |
| $\mu$ | Birth rate |
| $\omega_{k}$ | Waning of HPV natural immunity (age k) |
| $\psi_{k,g}$ | Waning of HPV vaccine-induced immunity (age k, genotype g) |
| $\gamma_{k,g}$ | Regression rate from infection to healthy state (age k, genotype g) |
| $\delta_{k,g}$ | Regression rate from low-grade CIN to healthy state (age k, genotype g) |
| $\alpha_{k,g}$ | Regression rate from low-grade CIN to infection (age k, genotype g) |
| $\beta_{k,g}$ | Regression rate from high-grade CIN to healthy state (age k, genotype g) |
| $\varepsilon_{k}$ | Cure rate of high-grade Cervical Intraepithelial Neoplasia treatment (age k) |
| $ê_{k}$ | Cure rate of low-grade Cervical Intraepithelial Neoplasia treatment (age k) |
| $\nu_{k}$ | Preadolescent vaccination coverage (age k) |
| $\lambda_{k,g}$ | Infection rate (age k, genotype g) |
| $\theta_{k}$ | Death rate due to other causes in women (age k) |
| $\tau_{k}$ | Effectiveness of the vaccine (age k) |
| $\rho_{k}$ | Vaccination coverage for catch-up component (age k) |
| $\eta_{k,g}$ | Progression rate from infection to low-grade CIN (age k, genotype g) |
| $\partial_{k,g}$ | Progression rate from infection to high-grade CIN (age k, genotype g) |
| $x_{g}$ | Effectiveness of the natural immunity (age k) |
| $\pi_{k,g}$ | Progression rate from low-grade CIN to high-grade CIN (age k, genotype g) |
| $\mathcal{F}_{k,g}$ | Progression rate from high-grade CIN to invasive cervical cancer (age k, genotype g) |
| $h$ | Progression rate from local cervical cancer to regional cervical cancer |
| $\mathcal{H}$ | Progression rate from regional cervical cancer to distant cervical cancer |
| $\psi_{k,g}$ | Regression rate from high-grade CIN to infection (age k, genotype g) |
| $\varpi_{k,g}$ | Regression rate from high-grade CIN to low-grade CIN (age k, genotype g) |
| $\mathfrak{D}$ | Symptomatic detection rate of local cervical cancer |
| $\mathbb{Q}$ | Symptomatic detection rate of regional cervical cancer |
| $\mathcal{L}$ | Symptomatic detection rate of distant cervical cancer |
| $\phi_{k}$ | Detection rate through screening for high-grade CIN (age k) |
| $æ_{k}$ | Detection rate through screening for low-grade CIN (age k) |
| $\mathcal{r}_{k}$ | Cure rate of local cervical cancer (age k) |
| $\mathfrak{y}_{k}$ | Cure rate of regional cervical cancer (age k) |
| $⋏_{k}$ | Death rate due to distant cervical cancer in women who do not receive treatment (age k) |
| $\ni_{k}$ | Death rate due to distant cervical cancer in women who receive treatment (age k) |
| $\mathcal{m}_{k}$ | Death rate due to local cervical cancer treatment (age k) |
| $\mathcal{n}_{k}$ | Death rate due to regional cervical cancer treatment (age k) |
| $los\_final$ | Proportion of loss to follow-up at three visits |
| $C$ | Proportion of vaccinated preadolescent girls/boy vaccination |
| $COV$ | Proportion of people given a catch-up component |
| $\varsigma_{k}$ | Screening coverage at age class k (age k) |
| $\varrho$ | Sensitivity of screening test |
| $\mathcal{g}$ | Proportion of women with local cervical cancer who accept the treatment |
| $\Omega$ | Proportion of women with regional cervical cancer who accept the treatment |
| $\mathcal{A}$ | Proportion of women with distant cervical cancer who accept the treatment |
| $\mathcal{o}$ | Male to female population ratio |
| $\mathcal{T}_{g}$ | Genotype-specific transmission probability |

| **Table 3: Model parameters: force of infection** | | | | | |
| --- | --- | --- | --- | --- | --- |
| **Age group** | **Male** | **Female** | **Adjusted ¶** | **Multiplier ‡** | **Source** |
| **Transmissibility per sexual partnership** | | | | | Calibrated |
| HPP 16 | 0.355 | 0.355 |  |  |  |
| HPV 18 | 0.40 | 0.40 |  |  |  |
| Other-HR HPV | 0.41 | 0.41 |  |  |  |
| Low-risk HPV | 0.39 | 0.39 |  |  |  |
| **Mean number of annual change of sexual partners among males and females** | | | | | [^36^](#_ENREF_36) |
| 12-13 | 0.222 | 0.071 | 1 | 2.48-4.43 |  |
| 14-15 | 0.673 | 0.283 | 1 |  |  |
| 15-19 | 3.794 | 2.48 | 0.7 |  |  |
| 20-24 | 5.802 | 2.442 | 0.7 |  |  |
| 25-29 | 2.957 | 1.728 | 0.7 |  |  |
| 30-34 | 2.113 | 0.971 | 0.7 |  |  |
| 35-39 | 1.323 | 0.842 | 0.7 |  |  |
| 40-44 | 1.323 | 0.842 | 1 |  |  |
| 45-49 | 0.662 | 0.421 | 1 |  |  |
| 50-54 | 0.662 | 0.421 | 2 |  |  |
| 55-64 | 0.331 | 0.211 | 2 |  |  |
| 65-74 | 0.166 | 0.106 | 3 |  |  |
| **Sexual mixing matrix** | | | | | [^19^](#_ENREF_19) |
| Same age | 0.6 | 0.6 |  |  |  |
| Different age | 0.4 | 0.4 |  |  |  |
| **¶** Adjusted values was applied to the force of infection model  **‡** Multiplier values ranged according to related-scenarios of annual incidence rate of cervical cancer | | | | | |

| **Table 4: Summary of input parameters for the model** | | | | | |
| --- | --- | --- | --- | --- | --- |
| **Parameters** | | | | **Baseline values*** | **Source** |
| **Progression** | | | | | |
| Healthy to infection † (-20 and +40%) | HPV-16 | 0.000175-0.003148 (0.0001426-0.00761) | | | Calibrated |
|  | HPV-18 | 0.0004-0.000789 (0.000102-0.00168) | | |  |
|  | Other HR HPV | 0.000206-0.004038 (0.0001703-0.00911) | | |  |
|  | LR HPV | 0.000958-0.018412 (0.00069-0.0537) | | |  |
| HPV DNA to CIN1‡ | HR-16 HPV | 0.005194-0.00901 | | | [^23^](#_ENREF_23) |
|  | HR-18 HPV | 0.002793-0.004845 | | |  |
|  | HR-other HPV | 0.007693-0.013345 | | |  |
|  | LR-HPV | 0.002397-0.001222 | | |  |
| Proportion (%) of women who transition directly from HPV DNA to CIN2,3 | HR-16 HPV | 0.64 | | |  |
|  | HR-18 HPV | 0.975 | | |  |
|  | HR-other HPV | 0.966 | | |  |
|  | LR-HPV | 0.98 | | |  |
| CIN 1 to CIN 2,3 ‡ | HR-16 HPV | 0.00951-0.012363 | | |  |
|  | HR-18 HPV | 0.0051-0.00663 | | |  |
|  | HR-other HPV | 0.00747-0.009711 | | |  |
|  | LR-HPV | 0.000149-0.000222 | | |  |
| CIN 2,3 to local cancer | HR-16 HPV | 0.000151-0.00906 | | |  |
|  | HR-18 HPV | 0.000264-0.01584 | | |  |
|  | HR-other HPV | 0.000199-0.01194 | | |  |
| Local to regional invasive cancer | | 0.0200 | | |  |
| Regional to distant invasive cancer | | 0.0250 | | |  |
| **Regression** | | | | |  |
| HPV DNA to Normal | HR-16 HPV | | 0.09089 | |  |
|  | HR-18 HPV | | 0.09089 | |  |
|  | HR-other HPV | | 0.09272 | |  |
|  | LR-HPV | | 0.09699 | |  |
| CIN 1 to normal ‡‡ | HR-16 HPV | | 0.03782 | |  |
|  | HR-18 HPV | | 0.03782 | |  |
|  | HR-other HPV | | 0.04575 | |  |
|  | LR-HPV | | 0.01708 | |  |
| CIN 2,3 to Normal §§ | HR-16 HPV | | 0.000798-0.000455 | |  |
|  | HR-18 HPV | | 0.003556-0.011938 | |  |
|  | HR-other HPV | | 0.002926-0.009823 | |  |
|  | LR-HPV | | 0.001904-0.006392 | |  |
| Other | | | | |  |
| Immunity (%) (HR-HPV types only) ¶¶ | HR-16 HPV | | 0.66 | |  |
|  | HR-18 HPV | | 0.86 | |  |
|  | HR-other HPV | | 0.59 | |  |
| Annual probability of symptom detection # | Local invasive cancer | | 0.33 | |  |
|  | Regional invasive cancer | | 0.60 | |  |
|  | Distant cancer | | 0.9 | |  |
| Proportion of cancer patient receiving the treatment | Local cancer | | 100% | | Calibrated |
|  | Regional cancer | | 87% | |  |
|  | Distant cancer | | 78% | |  |
| Age-specific 5-year survival proportion after diagnosis and treatment (%) £ | Local cancer | | 0.29-71% | | Calibrated |
|  | Regional cancer | | 0.24-78% | |  |
| Age-specific monthly probability of death | Complication of local cancer treatment | | 0.012-0.037 | | Calibrated |
|  | Complication of regional cancer treatment | | 0.0098-0.028 | |  |
|  | Distant cancer (rate) | | 0.28-0.83 | |  |
| Age-specific all cause death rates per person per year | Female | | 0,00106-0,4122 | | [^37^](#_ENREF_37) |
|  | Male | | 0.001-0.47 | |  |
| * Baseline values are monthly age-specific probabilities, unless otherwise noted  † The transition from healthy state to infection is a force of infection derived from the number of sexual partner change, HPV type-specific transmissibility.  ‡ HPV, human papillomavirus; CIN, cervical intraepithelial neoplasia; HR, high risk; LR, low risk  ‡‡ 70% of women with CIN 1 regress to normal, 30% to HPV.  §§ 70% of women with CIN2,3 regress to normal, 15% to HPV, 15% to CIN 1.  ¶¶ Immunity represents the degree to protection each woman faces against future type-specific infection after infection after first infection and clearance. The immunity was assumed to be lifelong.  # The annual probability of symptom detection corresponds to 15% for local cancer and 85% for advanced cancer  £ Age-specific survival proportion was calibrated, based on a mortality rate estimated by Globocan.[^3^](#_ENREF_3) | | | | | |

| **Table 5: Vaccination, screening, compliance and costing parameters** | | | | | | | |
| --- | --- | --- | --- | --- | --- | --- | --- |
| **Items** | | | | **Value** | **Source** # | | |
| Vaccination coverage | | | | 70% (30-80%) | Assumption | | |
| Vaccine efficacy against HPV type 16 and 18 infection | | | | 100% (30-100%) | [^38^](#_ENREF_38) | |  |
| Wane of vaccine or natural immunity | | | | Lifelong (10 years to lifelong) | Assumption | | |
| Routine cervical cytology coverage | | | | 5.2% every 3 years | [^3^](#_ENREF_3) | | |
| Cervical cytology | | Sensitivity | | 59% | [^26^](#_ENREF_26) | | |
|  |  | Specificity | | 94% |  | | |
| Colposcopy | | Sensitivity | | 96% | [^39^](#_ENREF_39) | | |
|  |  | Specificity | | 48% |  | | |
| True positive women received treatment† | | | | 55% |  | | |
| Proportion of cure for LEEP | | High-grade CIN | | 96.7% | [^24^](#_ENREF_24) | | |
| Proportion of cure for simple hysterectomy (Any CIN) | | | | 99% | [^40^](#_ENREF_40) | | |
| Lost to follow-up per visit | | | | 15% | Assumption | | |
| Cost of conventional cervical cytology‡§ | | | | 48.27 | Personal communication ¶ | | |
| Cost of colposcopy‡ | | | | 17.87 | Personal communication ¶ | | |
| Cost of biopsy‡§ | | | | 45.69 |  |  |  |
| Cost of LEEP‡ |  | | | 120.40 | Personal communication ¶ | | |
| Cost of simple hysterectomy ‡ | | | | 1188.59 |  |  |  |
| Treatment cost of local cancer ‡ | | | | 745.57 (372.79-1491.15) | | [^30^](#_ENREF_30) | |
| Treatment cost of regional cancer ‡ | | | | 845.68 (422.85-1691.36) | | [^30^](#_ENREF_30) | |
| Treatment cost of distant cancer ‡ | | | | 845.68 (422.85-1691.36) | | [^30^](#_ENREF_30) | |
| Cost of vaccination * | | | | 42.59 | | [^31^](#_ENREF_31) | |
| Disability weighting for diagnosis and primary therapy | | | Local cancer | 0.2 (0.199-0.411) | | [^32^](#_ENREF_32) | |
|  |  |  | Regional cancer | 0.411 (0.199-0.411) | |  |  |
| Disability weighting for distant cancer | | | | 0.683 (0.356-0.683) | |  |  |
| Discount rate‡‡ | | | | 3% (0-5%) | | [^1^](#_ENREF_1) | |
| **Note:**  # Databases of the Department of Health Insurance, Pathology Centre, and Department of National Immunisation Programme, Ministry of Health, Lao PDR.  † A true positive result of cervical cytology was defined as a high-grade CIN, considering 15% loss to follow-up over the three expected visits (for screening, diagnostic test, and treatment).  § In the Vientiane Capital, four pathology technicians work together and can prepare a total of 50 smear slides for conventional cervical cytology per day. They can also prepare 10 histology slides per day. A pathologist needs 20 to 35 minutes for a cytology and histology examination.  ‡ Cost is unit price per person, 2013 International dollars exchange using purchasing power parity (PPP) exchange rate (1 I$ = 2 694.27 kips)[^29^](#_ENREF_29) and the price of cancer treatment was adjusted from 2005 to 2014 using consumer price index (77.33 in 2005 and 122.52 in 2014).[^41^](#_ENREF_41)  * Vaccination costs include programmatic cost and vaccine cost, 29.09 and 13.5 international dollars per three doses, respectively. We communicated with WHO in Lao PDR to get the programmatic cost of vaccination. Vaccine costs varied from 13.5-300 international dollars per three doses.  All costs varied +/- 75% with the exception of cervical cancer treatment  ¶ A head of department of health insurance, Ministry of health, Lao PDR was contacted for costing data  ‡‡ Discount rate for cost is maximum of 6% and 5% for DALYs | | | | | | | |

| **Table 6: Calibration target** | | | | | |
| --- | --- | --- | --- | --- | --- |
| **Calibration target** | | **Source** | **Calibration target** | | **Source** |
| **Female population¶** | | [^41^](#_ENREF_41) | **Annual incidence rates of invasive cervical cancer per 100,000** | | [^3^](#_ENREF_3) |
| 0- <5 | 44196 |  | 15-39 | 5.2 |  |
| 5- <10 | 40488 |  | 40-44 | 26.9 |  |
| 10 - <15 | 27947 |  | 45-49 | 33.3 |  |
| 15 - < 20 | 31402 |  | 50-54 | 37.1 |  |
| 20 - < 25 | 38205 |  | 55-59 | 36.9 |  |
| 25 - < 30 | 48941 |  | 60-64 | 34.8 |  |
| 30 - < 35 | 45627 |  | 65-69 | 33.7 |  |
| 35 - < 40 | 32125 |  | 70-74 | 30.5 |  |
| 40 - < 45 | 26762 |  | >74 | 29 |  |
| 45 - < 50 | 21895 |  | **Annual mortality of invasive cervical cancer per 100,000** | |  |
| 50 - < 55 | 17307 |  | 15-39 | 1.2 |  |
| 55 - < 60 | 12766 |  | 40-44 | 9.6 |  |
| 60 - < 65 | 8251 |  | 45-49 | 14.2 |  |
| 65 - < 70 | 5930 |  | 50-54 | 19.8 |  |
| 70 - < 75 | 4152 |  | 55-59 | 23.9 |  |
| 75+ | 6119 |  | 60-64 | 27.9 |  |
| **Distribution of HPV types among women with cancer, Thai data** | | [^21^](#_ENREF_21) | 65-69  70-74  >74 | 31.8  35.6  39.4 |  |
| HPV1618 | 75.1 |  |  |  |  |
| Other-HR HPV‡ | 24.9 |  |  |  |  |
| ¶ The proportion of male population to female population is 0.948  ‡ HPV, human papillomavirus | | | | | |

| **Table 7: Costing parameters for screening** | | | |
| --- | --- | --- | --- |
| **Option** | **Items** | **Unit price (2013 I$)** | **Source** |
| Conventional cervical cytology | Administration ‡ | 14.48 | Personal communication with a head of department of health insurance. Ministry of health, Lao PDR |
|  | Medical staff ¶ | 3.39 |  |
|  | Cervical cytology laboratory equipment | 11.20 | Personal communication with a head of department of Pathology center, University of Health Science, Ministry of Health, Lao PDR |
|  | Laboratory staff | 3.54 |  |
|  | **Total** | 32.61 |  |
| Colposcopy | Administration ‡ | 14.48 | Personal communication with a head of department of health insurance. Ministry of health, Lao PDR |
|  | Medical staff ¶ | 3.39 |  |
|  | **Total** | 17.87 |  |
| **Note:**  ‡ Administration includes general and medical administration. General administration includes electricity, water and transportation supplies, and other office materials and staffs. Medical administration included training support and aids, and some medical equipment.  ¶ Monthly salary also includes incentives, gasoline, and overtime pay. Salary per hour = salary per day/8; Salary per day =(monthly salary x 12 months) / (52 weeks x 5 working days).   - Monthly average salary of a gynaecologist is I$1 303 - Monthly average salary of a nurse is I$ 736 - Monthly average salary of a pathologist is I$ 992 - Monthly average salary of a pathology technician is I$ 717   International dollars exchange rate using 2013 purchasing power parity (PPP) exchange rate (1 I$ = 2 694.27 Lao kip).[^29^](#_ENREF_29) | | | |

| **Table 8: Details of laboratory costs** | | | |
| --- | --- | --- | --- |
| **Item** | **Sub-item** | **Unit price (dollar)** | **Source** |
| Conventional cervical cytology | Lab administration ‡ | 0.01 | Personal communication with a head of department of Pathology center, University of Health Science, Ministry of Health, Lao PDR |
|  | Lab equipment # | 11.20 |  |
|  | Lab staff ¶ | 3.54 |  |
|  | **Total** | 14.75 |  |
| Histology | Lab administration ‡ | 14.48 |  |
|  | Lab equipment * | 15.47 |  |
|  | Lab staff ¶ | 15.74 |  |
|  | **Total** | 45.69 |  |
| **Note:**  # Consumable items included brushes, cover glasses, Malinol, Gill's hematoxylin, OG-6, EA-50, masks, xylene, ethanol, slides. LBC prep set, LBC liquid were added for Thin-Prep.  * Consumable items included Formaline, hematocyline , eosine, paraffine, cassette, cyline, absolute alcohol, acetone, malinone.  ‡ Lab administration costs were retrieved from general administration allocated to laboratory in hospital per sample.  ¶ This included both technical staff and pathologist cost. Each cost is calculated by multiplying time spending to procedure with labour cost per hour.  International dollars exchange using 2013 purchasing power parity (PPP) exchange rate (1 I$ = 2 694.27 Lao kip).[^29^](#_ENREF_29) | | | |

| **Table 9 Costing of precancerous treatment** | | | |
| --- | --- | --- | --- |
| **Item** | **Sub-item** | **Unit price (dollar)** | **Source** |
| LEEP | Administration ‡ | 27.66 | Personal communication with a head of department of health insurance, Ministry of health, Lao PDR |
|  | Drug and equipment cost * | 57.05 |  |
|  | Labour cost ‡‡ | 35.68 |  |
|  | **Total** # | 120.40 |  |
| Hysterectomy | Administration † | 64.63 |  |
|  | Drug and medical equipment cost † | 204.23 |  |
|  | Labour cost † | 76.96 |  |
|  | **Subtotal** | 345.82 |  |
|  | Hospitalisation cost in 7 days § | 842.78 |  |
|  | **Total** # | 1188.59 |  |
| Cancer treatment §§ | Treatment cost of local cancer | 745.57 (372.79-1491.15) | [^30^](#_ENREF_30) |
|  | Treatment cost of regional cancer | 845.68 (422.85-1691.36) |  |
|  | Treatment cost of distant cancer | 845.68 (422.85-1691.36) |  |
| **Note:**  ‡ Administration included general and medical administration. General administration included electricity, water and transportation supplies, and other office materials and staffs. Medical administration included training support and aids, some medical equipment; inpatient for loop electrosurgical excision procedure (LEEP).  ‡‡ Labour cost was calculated by multiplying the wage rate per hour by the time spent to provide treatment  * Drug and equipment cost consists of the average cost per patient of in- and out-patient clinics.  † Due to lack of data specific to obstetric surgery, administration, drug and medical equipment and labour cost of hysterectomy an average cost of a surgery case at the department of gynaeco-obstetrics was used.  § Hospitalisation cost consists of the average cost of hospitalisation per day at the department of gynaeco-obstetrics. We assumed that a patient was hospitalised for seven days  ≠ Total cost did not include the cost of follow-up for precancerous lesions because, according to experts, patients are lost to follow-up.  §§ Cost is unit price per person, 2013 International dollars exchange using purchasing power parity (PPP) exchange rate (1 I$ = 2 694.27 Lao kip)[^29^](#_ENREF_29) and the price of cancer treatment was adjusted from 2005 to 2014 using the consumer price index (77.33 in 2005 and 122.52 in 2014).[^41^](#_ENREF_41) | | | |

**Results**

**Figure 2: model calibration to age specific demographic distribution in female and male of Vientiane capital**


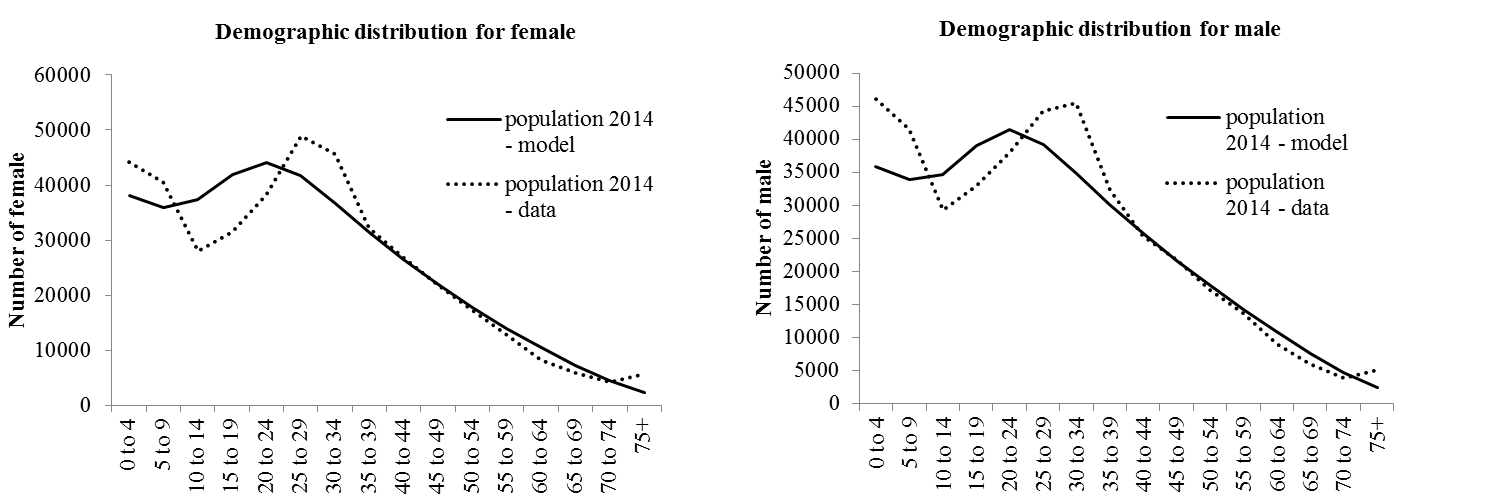


**Figure 3: Reduction of force infection for HPV type 16 over 100 years simulation**


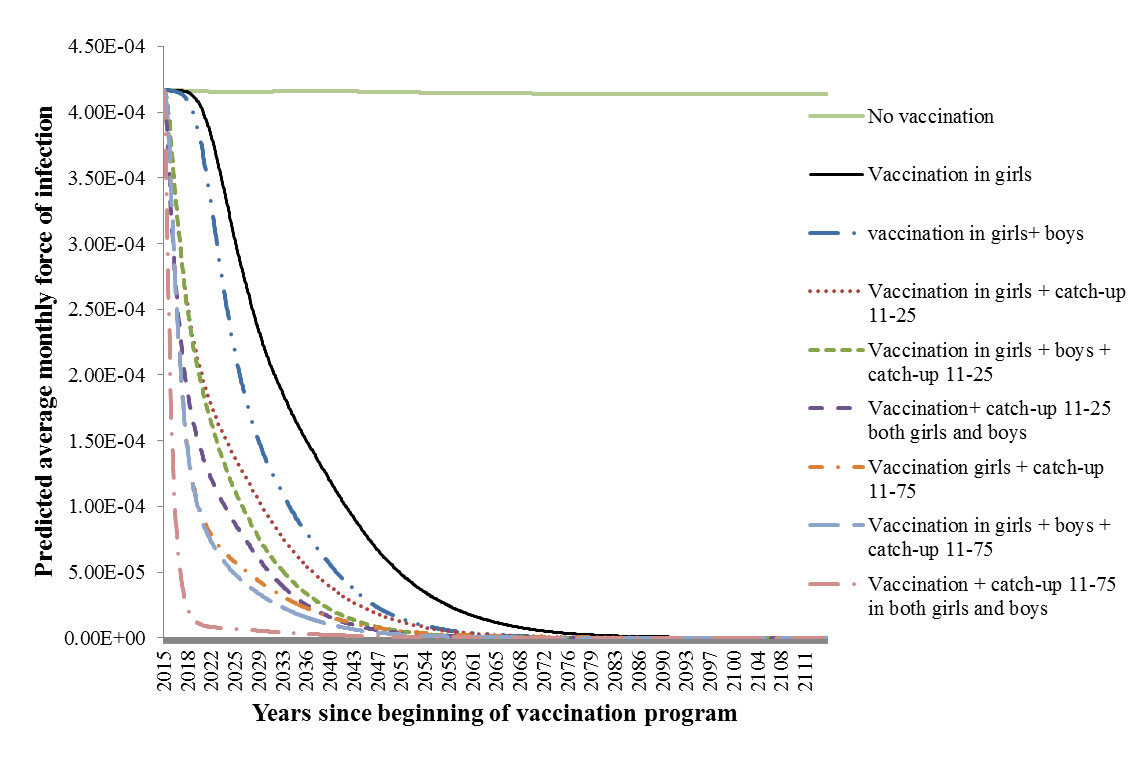


**Figure 4: Reduction of the prevalence of HPV type 16 and 18 during 100 years simulation**


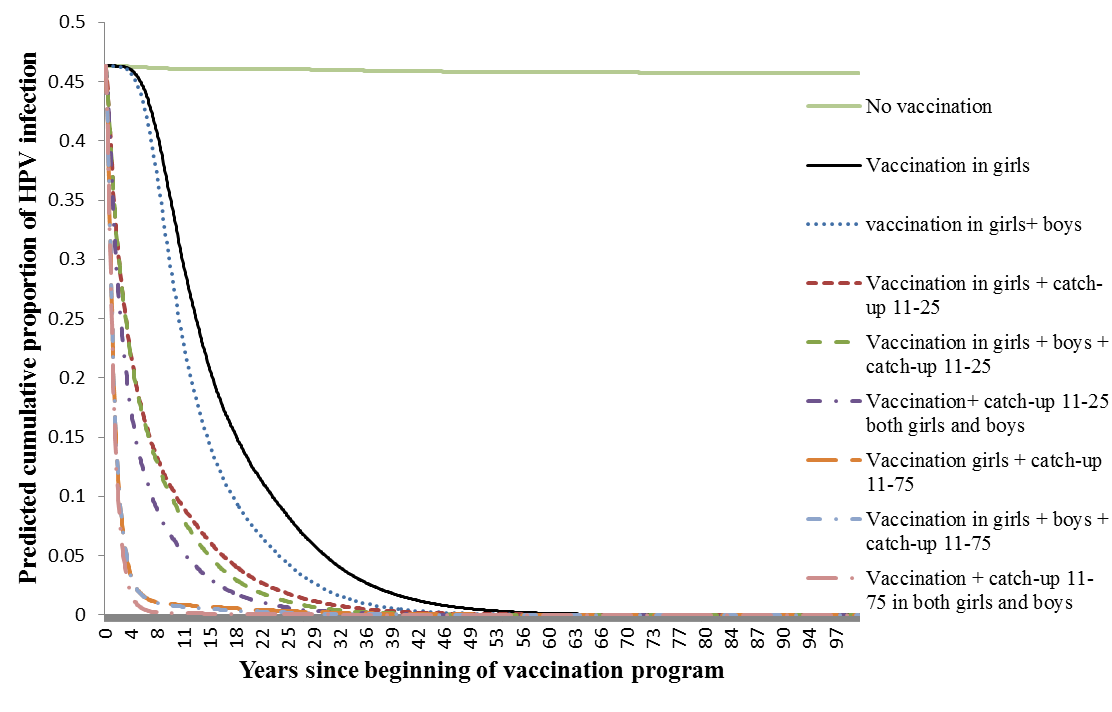


| **Table 10 The cost-effectiveness of HPV vaccination against cervical cancer due to all high-risk HPV** | | | | | | |
| --- | --- | --- | --- | --- | --- | --- |
| Number | Options | Total cost per 1000 women | Cancer due to other high-risk HPV per 1000 women | Cancer increase (%) | DALY averted per 1000 women | ICER (DALY) |
|  | No vaccination with current screening | 4716 | 1.36 | Ref | Ref | - |
|  | 10 year old girls | 21824 | 1.40 | 2.2 | 30.7 | 557 |
|  | 10 year old girls+catch-up girls aged 11-25 years old | 28033 | 1.39 | 2.2 | 34.7 | 1547 |
|  | 10 year old girls+boys | 38256 | 1.39 | 2.0 | 32.2 | D |
|  | 10 year old girls+catch-up girls aged 11-75 years old | 39285 | 1.39 | 2.0 | 36.7 | 5774 |
|  | 10 year old girls+boys+catch-up girls aged 11-25 years old | 44489 | 1.39 | 2.0 | 35.0 | D |
|  | 10 year old girls+boys+catch-up girls and boys aged 11-25 years old | 50436 | 1.39 | 1.9 | 35.5 | D |
|  | 10 year old girls+boys+catch-up girls aged 11-75 years old | 55746 | 1.39 | 1.9 | 36.7 | D |
|  | 10 year old girls+boys+catch-up girls and boys aged 11-75 years old | 72949 | 1.39 | 1.8 | 36.9 | 168320 |
| **Note:** The incremental cost of effectiveness ratio expressed as cancer prevented or DALY averted is listed in order of increasing cost. In the non-dominant strategy, the ICER was calculated by devising different cost to different effectiveness. The D refers to strong dominance, which is expressed as higher cost, but lower effectiveness than alternative options. | | | | | | |

| **Table 11: Univariate sensitivity analyses of parameters influencing the incremental cost per DALY averted by vaccination strategies** | | | | | | | | | | | | | |
| --- | --- | --- | --- | --- | --- | --- | --- | --- | --- | --- | --- | --- | --- |
| Parameters | | | | | Baseline | Girl vaccination only | Girl + catch-up girls aged up to 25 years old | Girl and boy | Girl + catch-up girls aged up to 75 years old | Girl and boy + catch-up girls aged up to 25 years old | Girl and boy + catch-up girls and boys aged up to 25 years old | Girl and boy + catch-up girls aged up to 75 years old | Girl and boy + catch-up girls and boys aged up to 75 years old |
| 10 consecutive cohorts vaccination | | | | Total cost | 4497 | 7676 | 10796 | 13810 | 16988 | 19951 | 25047 | 28236 | 45437 |
|  |  |  |  | DALY averted | Ref | 18.4 | 25.6 | 32.1 | 33.6 | 34.4 | 36.0 | 36.4 | 36.9 |
|  |  |  |  | ICER | - | 173 | 428 | 467 | 2112 | 3530 | 3359 | 7443 | 32586 |
| Time horizon (years) | 30 | | | Total cost | 6353 | 29627 | 46483 | 51684 | 68567 | 76576 | 76869 | 98960 | 145371 |
|  |  | | | DALY averted | Ref | 2.5 | 6.9 | 3.6 | 7.2 | 7.7 | 10.7 | 10.7 | 10.9 |
|  |  | | | ICER | - | ED | ED | D | ED | ED | 6608 | ED | 306225 |
|  | 50 | | | Total cost | 6482 | 30695 | 42922 | 53778 | 65065 | 66048 | 71887 | 88200 | 122050 |
|  |  | | | DALY averted | Ref | 11.5 | 18.4 | 13.7 | 22.0 | 18.8 | 19.3 | 22.1 | 22.3 |
|  |  | | | ICER | - | ED | 1983 | D | 6043 | D | D | ED | 195543 |
| Initial age (years) | 11 | | | Total cost | 4497 | 21393 | 27237 | 37636 | 38489 | 43504 | 49100 | 54761 | 71612.9 |
|  |  |  |  | DALY averted | Ref | 31.4 | 35.1 | 32.9 | 37.0 | 35.3 | 35.9 | 37.0 | 37.2 |
|  |  |  |  | ICER | - | 537 | 1614 | D | 5791 | D | D | ED | 16935 |
|  | 12 | | | Total cost | 4497 | 21197 | 26674 | 37263 | 37925 | 42762 | 48004 | 54020 | 70517 |
|  |  |  |  | DALY averted | Ref | 31.8 | 35.0 | 33.2 | 37.0 | 35.3 | 35.8 | 37.0 | 37.2 |
|  |  |  |  | ICER | - | 526 | 1682 | D | 5738 | D | D | ED | 16733 |
|  | 13 | | | Total cost | 4497 | 21197 | 26297 | 37092 | 37546 | 42216 | 47099 | 53472 | 69611 |
|  |  |  |  | DALY averted | Ref | 31.8 | 34.9 | 33.3 | 37.0 | 35.3 | 35.8 | 37.0 | 37.2 |
|  |  |  |  | ICER | - | 526 | 1616 | D | 5526 | D | D | ED | 16347 |
| Vaccination coverage (%) | | | 30 | Total cost | 4497 | 7470 | 8544 | 10440 | 10553 | 11540 | 12606 | 13563 | 16692 |
|  |  |  |  | DALY averted | Ref | 25.8 | 30.4 | 29.7 | 33.4 | 32.4 | 33.7 | 34.5 | 35.8 |
|  |  |  |  | ICER | - | 115 | 234 | D | 676 | D | ED | ED | 2558 |
|  |  |  | 50 | Total cost | 4497 | 11683 | 14290 | 18699 | 19073 | 21334 | 23868 | 26125 | 33485 |
|  |  |  |  | DALY averted | Ref | 29.5 | 33.8 | 31.7 | 36.1 | 34.5 | 35.3 | 36.3 | 36.9 |
|  |  |  |  | ICER | - | 244 | 603 | D | 2086 | D | D | ED | 18015 |
|  |  |  | 80 | Total cost | 4497 | 34017 | 44731 | 62210 | 64065 | 72946 | 83154 | 92285 | 121781 |
|  |  |  |  | DALY averted | Ref | 31.6 | 35.4 | 32.9 | 37.2 | 35.5 | 36.0 | 37.2 | 37.3 |
|  |  |  |  | ICER | - | 934 | 2848 | D | 10590 | D | D | ED | 577160 |
| Vaccine cost per dose (I$) | | 30 | | Total cost | 4497 | 52844 | 70372 | 98838 | 101940 | 116389 | 133044 | 147962 | 196064 |
|  |  |  |  | DALY averted | Ref | 31.1 | 35.1 | 32.6 | 37.0 | 35.3 | 35.9 | 37.0 | 37.2 |
|  |  |  |  | ICER | - | 1555 | 4403 | D | 16384 | D | D | D | 520626 |
|  |  | 50 | | Total cost | 4497 | 77350 | 103756 | 146529 | 151258 | 172959 | 198012 | 220465 | 292802 |
|  |  |  |  | DALY averted | Ref | 31.1 | 35.1 | 32.6 | 37.0 | 35.3 | 35.9 | 37.0 | 37.2 |
|  |  |  |  | ICER | - | 2343 | 6633 | D | 24654 | D | D | D | 782919 |
|  |  | 100 | | Total cost | 4497 | 138614 | 187215 | 265759 | 274553 | 314382 | 360431 | 401722 | 534647 |
|  |  |  |  | DALY averted | Ref | 31.1 | 35.1 | 32.6 | 37.0 | 35.3 | 35.9 | 37.0 | 37.2 |
|  |  |  |  | ICER | - | 4312 | 12208 | D | 45329 | D | D | D | 1438653 |
| Incidence of cervical cancer (%) | | - 20 | | Total cost | 4429 | 21559 | 27777 | 37994 | 39036 | 44234 | 50182 | 55498 | 72700 |
|  |  |  |  | DALY averted | Ref | 28.1 | 31.7 | 29.4 | 33.4 | 31.9 | 32.4 | 33.4 | 33.6 |
|  |  |  |  | ICER | - | 609 | 1746 | D | 6612 | D | D | D | 206258 |
|  |  | +40 | | Total cost | 4745 | 21746 | 33943 | 38163 | 45172 | 50397 | 53363 | 61631 | 78839 |
|  |  |  |  | DALY averted | Ref | 41.9 | 47.7 | 44.0 | 50.3 | 48.0 | 48.3 | 50.4 | 50.5 |
|  |  |  |  | ICER | - | 406 | 2095 | D | 4306 | D | D | D | 180744 |
| Duration of natural immunity (10 years) | | | | Total cost | 5011 | 21883 | 28016 | 38286 | 39214 | 44465 | 47426 | 55673 | 72886 |
|  |  |  |  | DALY averted | Ref | 55.5 | 62.8 | 58.3 | 66.5 | 63.3 | 63.9 | 66.6 | 66.8 |
|  |  |  |  | ICER | - | 304 | 835 | D | 3043 | D | D | ED | 99721 |
| Duration of vaccine protection (10 years) | | | | Total cost | 4497 | 21695 | 27899 | 38089 | 39135 | 44319 | 50256 | 55570 | 72754 |
|  |  |  |  | DALY averted | Ref | 22.8 | 28.3 | 28.6 | 32.0 | 32.1 | 33.3 | 34.5 | 35.6 |
|  |  |  |  | ICER | - | 754 | 1131 | ED | 3037 | ED | ED | 6574 | 15622 |
| Disability weighting for local cancer treatment (0.411) | | | | Total cost | 4497 | 21599 | 27807 | 38030 | 39059 | 44263 | 50210 | 55520 | 72723 |
|  |  |  |  | DALY averted | Ref | 31.1 | 35.1 | 32.6 | 37.0 | 35.4 | 35.9 | 37.1 | 37.2 |
|  |  |  |  | ICER | - | 549 | 1558 | D | 5835 | D | D | ED | 168320 |
| 30% efficacy | | | | Total cost | 4497 | 21643 | 27856 | 38061 | 39102 | 44300 | 50239 | 55557 | 72743 |
|  |  |  |  | DALY averted | Ref | 28.3 | 32.8 | 31.0 | 35.3 | 33.8 | 34.8 | 35.7 | 36.5 |
|  |  |  |  | ICER | - | 605 | 1393 | D | 4411 | D | D | ED | 28034 |
| Cost of cancer treatment | | Lower bound | | Total cost | 4218 | 21450 | 27699 | 37895 | 38979 | 44158 | 50112 | 55440 | 72645 |
|  |  |  |  | DALY averted | Ref | 31.1 | 35.1 | 32.6 | 37.0 | 35.3 | 35.9 | 37.0 | 37.2 |
|  |  |  |  | ICER | Ref | 554 | 1570 | D | 5854 | D | D | D | 186215 |
|  |  | Upper bound | | Total cost | 5053 | 21898 | 28023 | 38302 | 39220 | 44474 | 50406 | 55681 | 72880 |
|  |  |  |  | DALY averted | Ref | 31.1 | 35.1 | 32.6 | 37.0 | 35.3 | 35.9 | 37.0 | 37.2 |
|  |  |  |  | ICER | Ref | 542 | 1538 | D | 5812 | D | D | D | 186183 |
| Discount rate (%) | | 0 | | Total cost | 2146 | 10134 | 16392 | 17744 | 24012 | 26982 | 27670 | 35291 | 52496 |
|  |  |  |  | DALY averted | Ref | 25 | 28.2 | 26.2 | 28.4 | 28.7 | 29.8 | 29.9 | 30 |
|  |  |  |  | ICER | - | 319 | 1947 | D | ED | ED | 7141 | ED | 171545 |
|  |  | 5 | | Total cost | 4497 | 21599 | 27807 | 38030 | 39059 | 44263 | 47230 | 55520 | 72723 |
|  |  |  |  | DALY averted | Ref | 46.3 | 52.2 | 48.4 | 54.9 | 52.5 | 52.9 | 55.0 | 55.2 |
|  |  |  |  | ICER | - | 369 | 1058 | D | 4097 | D | D | ED | 124047 |
| **Note:**  The total cost and DALY averted are per 1 00 women.  ICER, the incremental cost-effectiveness ratio expressed as DALY averted is listed in order of increasing cost. In the non-dominant strategy, the ICER was calculated by devising different cost to different effectiveness. The D refers to strong dominance, which is expressed as higher cost, but lower effectiveness than alternative options. | | | | | | | | | | | | | |

| **Table 12: The cost-effectiveness of vaccination strategies for Vientiane province (smaller population size)** | | | | | | | | | |
| --- | --- | --- | --- | --- | --- | --- | --- | --- | --- |
| Number | Options | Total cost per 1000 women | Cancer averted per 1 000 women (Number) | Cancer averted per 1 000 women  (%) | DALY averted per 1000 women | CER (cancer averted) | CER (DALY averted) | ICER (cancer averted) | ICER (DALY averted) |
|  | No vaccination with current screening | 2203 | Ref | Ref | Ref | - | - | - | - |
|  | 10 year old girls | 15403 | 1.7 | 86.8 | 19.3 | 9151 | 4257 | 7842 | 685 |
|  | 10 year old girls+catch-up girls aged 11-25 years old | 17848 | 1.8 | 92.4 | 20.7 | 9957 | 8104 | 22381 | 1727 |
|  | 10 year old girls+boys | 21436 | 1.8 | 95.0 | 21.3 | 11635 | 13398 | 71811 | 5957 |
|  | 10 year old girls+catch-up girls aged 11-75 years old | 28017 | 1.7 | 89.1 | 19.8 | 16218 | 9153 | D | D |
|  | 10 year old girls+boys+catch-up girls aged 11-25 years old | 30470 | 1.8 | 92.8 | 20.8 | 16928 | 14450 | D | D |
|  | 10 year old girls+boys+catch-up girls and boys aged 11-25 years old | 32811 | 1.8 | 93.5 | 21.0 | 18087 | 16938 | D | D |
|  | 10 year old girls+boys+catch-up girls aged 11-75 years old | 34060 | 1.8 | 95.1 | 21.3 | 18472 | 21566 | D | D |
|  | 10 year old girls+boys+catch-up girls and boys aged 11-75 years old | 40003 | 1.8 | 95.3 | 21.4 | 21650 | 26166 | 3495444 | 117542 |
| **Note:** The incremental cost of effectiveness ratio expressed as cancer prevented or DALY averted is listed in order of increasing cost. In the non-dominant strategy, the ICER was calculated by devising different cost to different effectiveness. The D refers to strong dominance, which is expressed as higher cost, but lower effectiveness than alternative options. | | | | | | | | | |

**References**

1. Adam T, Baltussen R, Tan Torres T, et al. Making Choices in Health: WHO Guide to Cost-Effectiveness Analysis. Geneva: World Health Organization; 2003. Available: <http://www.who.int/choice/publications/p_2003_generalised_cea.pdf>. Accessed: 03 February 2013.

2. Population: population by sex and age group in 2010-2011. Vientiane Capital: Lao statistics Bureau; 2011. Available: <http://www.nsc.gov.la/index.php?option=com_content&view=article&id=37:population&catid=6&Itemid=38>. Accessed: 22 March 2013.

3. Bruni L, Barrionuevo-Rosas L, Serrano B, et al. Human Papillomavirus and Related Diseases in Laos. ICO Information Centre on HPV and Cancer (HPV Information Centre). 2014.

4. Brennan A, Chick SE, Davies R. A taxonomy of model structures for economic evaluation of health technologies. *Health Econ* 2006; **15**(12): 1295-310.

5. Summary report on HPV and cervical cancer statistics in Laos 2010. WHO/ICO Information Centre on HPV and Cervical Cancer (HPV Information Centre). Geneva: HPV Information Centre, 2012.

6. Guidelines for the Economic Evaluation of Health Technologies: Canada. Canadian Agency for Drugs and Technologies in Health (cadth). 3rd ed. Ottawa, ON: CADTH; 2006.

7. Health Service Delivery Profile: Lao PDR; 2012. Minstry of Health and WHO. Available: <http://www.wpro.who.int/health_services/service_delivery_profile_laopdr.pdf>. Accessed: 21 May 2014

8. Lao People's Democratic Republic. United Nations, 2013. Available: <http://data.un.org/CountryProfile.aspx?crName=Lao%20People%27s%20Democratic%20Republic>. Accessed: 19 November 2013.

9. Kim JJ, Andres-Beck B, Goldie SJ. The value of including boys in an HPV vaccination programme: a cost-effectiveness analysis in a low-resource setting. *British journal of cancer* 2007; **97**(9): 1322-8.

10. Jit M, Choi YH, Edmunds WJ. Economic evaluation of human papillomavirus vaccination in the United Kingdom. *BMJ (Clinical research ed)* 2008; **337**: a769.

11. Barnabas RV, Laukkanen P, Koskela P, Kontula O, Lehtinen M, Garnett GP. Epidemiology of HPV 16 and cervical cancer in Finland and the potential impact of vaccination: mathematical modelling analyses. *PLoS medicine* 2006; **3**(5): e138.

12. Richart RM. A modified terminology for cervical intraepithelial neoplasia. *Obstetrics and gynecology* 1990; **75**(1): 131-3.

13. Gupta N, Srinivasan R, Rajwanshi A. Functional biomarkers in cervical precancer: an overview. *Diagnostic cytopathology* 2010; **38**(8): 618-23.

14. Moscicki AB, Schiffman M, Kjaer S, Villa LL. Chapter 5: Updating the natural history of HPV and anogenital cancer. *Vaccine* 2006; **24 Suppl 3**: S3/42-51.

15. Borruto F, Comparetto C. Treatment, Follow-up, and Prevention of Papillomavirus Infection and Cervical Cancer. In: Borruto F, Ridder MD, eds. HPV and Cervical Cancer. New York: Springer Science+Business Media; 2012: 273-318.

16. Holowaty P, Miller AB, Rohan T, To T. Natural history of dysplasia of the uterine cervix. *Journal of the National Cancer Institute* 1999; **91**(3): 252-8.

17. Benedet JL, Bender H, Jones H, 3rd, Ngan HY, Pecorelli S. FIGO staging classifications and clinical practice guidelines in the management of gynecologic cancers. FIGO Committee on Gynecologic Oncology. *International journal of gynaecology and obstetrics: the official organ of the International Federation of Gynaecology and Obstetrics* 2000; **70**(2): 209-62.

18. Pecorelli S, Zigliani L, Odicino F. Revised FIGO staging for carcinoma of the cervix. *International journal of gynaecology and obstetrics: the official organ of the International Federation of Gynaecology and Obstetrics* 2009; **105**(2): 107-8.

19. Lao PDR: Lao Social Indicator Survey (LSIS). Vientiane: Ministry of Health; 2012.

20. Sychareun V, Phengsavanh A, Hansana V, et al. Predictors of premarital sexual activity among unmarried youth in Vientiane, Lao PDR: the role of parent-youth interactions and peer influence. *Global public health* 2013; **8**(8): 958-75.

21. Bruni L, Barrionuevo-Rosas L, Serrano B, et al. Human Papillomavirus and Related Diseases in Thailand. ICO Information Centre on HPV and Cancer (HPV Information Centre). 2014.

22. de Sanjose S, Quint WG, Alemany L, et al. Human papillomavirus genotype attribution in invasive cervical cancer: a retrospective cross-sectional worldwide study. *The lancet oncology* 2010; **11**(11): 1048-56.

23. Kim JJ, Kuntz KM, Stout NK, et al. Multiparameter calibration of a natural history model of cervical cancer. *American journal of epidemiology* 2007; **166**(2): 137-50.

24. Suprasert P, Panyaroj W, Kietpeerakool C. Recurrent rates with cervical intraepithelial neoplasia having a negative surgical margin after the loop electrosurgical excision procedure in Thailand. *Asian Pacific journal of cancer prevention : APJCP* 2009; **10**(4): 587-90.

25. WHO. Comprehensive cervical cancer control : a guide to essential practice-– 2nd ed. 2014. Available: <http://apps.who.int/iris/bitstream/10665/144785/1/9789241548953_eng.pdf>. Accessed: 09 November 2014.

26. Chen C, Yang Z, Li Z, Li L. Accuracy of several cervical screening strategies for early detection of cervical cancer: a meta-analysis. *International journal of gynecological cancer : official journal of the International Gynecological Cancer Society* 2012; **22**(6): 908-21.

27. Macey R, George O, Zahnley T. Berkeley Madonna (Version 8.3.18). California: University of California; 2010. Available: http://www.berkeleymadonna.com/download.html.

28. World Population Prospects: The 2012 Revision, DVD Edition. United Nations; 2013. Available: <http://esa.un.org/unpd/wpp/Excel-Data/population.htm>. Accessed: 15 October 2013.

29. PPP conversion factor, GDP (LCU per international $): World Development Indicators. World Bank, International Comparison Program database; 2014. Available: <http://data.worldbank.org/indicator/PA.NUS.PPP>. Accessed: 17 March 2015.

30. Goldie SJ, O'Shea M, Campos NG, Diaz M, Sweet S, Kim SY. Health and economic outcomes of HPV 16,18 vaccination in 72 GAVI-eligible countries. *Vaccine* 2008; **26**(32): 4080-93.

31. WHO. 6th Global Meeting on Implementing New and Under-utilized Vaccines, 15-17 May 2012. Geneva: WHO, 2013. Available: <http://www.who.int/nuvi/2012_meeting_summary_introduction/en/index.html>. Accessed: 25 June 2014.

32. Salomon JA, Vos T, Hogan DR, et al. Common values in assessing health outcomes from disease and injury: disability weights measurement study for the Global Burden of Disease Study 2010. *Lancet* 2012; **380**(9859): 2129-43.

33. Drummond MF, Sculpher MJ, Torrance GW, O'Brien BJ, Stoddart GL. Methods for the economic evaluation of health care programmes. 3rd ed ed. Oxford ; New York: Oxford University Press; 2005.

34. WHO Commission on Macroeconomics and Health. Macroeconomics and health: Investing in health for economic development. Geneva, 2001. Available: <http://whqlibdoc.who.int/publications/2001/924154550x.pdf>. Accessed: 07 June 2014

35. GDP per capita, PPP (current international $): World Development Indicators. World Bank, 2014. Available: <http://data.worldbank.org/indicator/NY.GDP.PCAP.PP.CD/countries?display=default>. Accessed: 17 March 2015.

36. Johnson AM, Mercer CH, Erens B, et al. Sexual behaviour in Britain: partnerships, practices, and HIV risk behaviours. *Lancet* 2001; **358**(9296): 1835-42.

37. Life tables for WHO Member States. Geneva: World Health Organization, 2012. Available: <http://apps.who.int/gho/data/node.imr.LIFE_0000000029?lang=en>. Accessed: 24 October 2014.

38. Bosch FX. Human papillomavirus: science and technologies for the elimination of cervical cancer. *Expert opinion on pharmacotherapy* 2011; **12**(14): 2189-204.

39. Mitchell MF, Schottenfeld D, Tortolero-Luna G, Cantor SB, Richards-Kortum R. Colposcopy for the diagnosis of squamous intraepithelial lesions: a meta-analysis. *Obstetrics and gynecology* 1998; **91**(4): 626-31.

40. Jones HW, 3rd. Cone biopsy and hysterectomy in the management of cervical intraepithelial neoplasia. *Bailliere's clinical obstetrics and gynaecology* 1995; **9**(1): 221-36.

41. Welcome to Lao Statistics Bureau. Vientiane Capital; 2014. Available: <http://www.nsc.gov.la/index.php?lang=en>. Date accessed: 24 October 2014.
